# LGR5 Is a Negative Regulator of Tumourigenicity, Antagonizes Wnt Signalling and Regulates Cell Adhesion in Colorectal Cancer Cell Lines

**DOI:** 10.1371/journal.pone.0022733

**Published:** 2011-07-28

**Authors:** Francesca Walker, Hui-Hua Zhang, Annalisa Odorizzi, Antony W. Burgess

**Affiliations:** Epithelial Biochemistry Laboratory, Ludwig Institute for Cancer Research, Parkville, Victoria, Australia; Université Paris-Diderot, France

## Abstract

**Background:**

LGR5 (Leucine-rich repeat-containing G-protein coupled receptor 5) is the most established marker for intestinal stem cells. Mouse models show that LGR5+ cells are the cells of origin of intestinal cancer, and LGR5 expression is elevated in human colorectal cancers, however very little is known about LGR5 function or its contribution to the stem cell phenotype and to colorectal cancer.

**Principal Findings:**

We have modulated the expression of LGR5 by RNAi (inhibitory RNAs) or overexpression in colorectal cancer cell lines. Paradoxically, ablation of LGR5 induces increased invasion and anchorage-independent growth, and enhances tumourigenicity in xenografts experiments. Conversely, overexpression of LGR5 augments cell adhesion, reduces clonogenicity and attenuates tumourigenicity. Expression profiling revealed enhanced wnt signalling and upregulation of EMT genes upon knockdown of LGR5, with opposite changes in LGR5 overexpressing cells. These findings suggest that LGR5 is important in restricting stem cells to their niche, and that loss of LGR5 concomitant with activated wnt signalling may contribute to the invasive phenotype of colorectal carcinomas.

## Introduction

The concept of cancer stem cells (CSCs: reviewed by [1]) arises from the heterogeneity of most solid tumours and their resistance to chemotherapeutic regimes: according to this concept, after treatment a residual population of drug-resistant cancer stem cells will survive and rapidly proliferate to re-establish the tumours ([2]). The relative resistance to chemotherapeutic drug has been attributed to dormancy or slow proliferation of CSCs, a characteristic shared with normal stem cells (see for example [3]). Support for the existence of human CSCs is the presence, within the tumours, of cellular subsets expressing proteins usually only found on stem cells and lost upon differentiation; these proteins have been used to enrich for the putative CSCs in different tumour types, and to prove that tumour cells enriched for these markers gives rise to tumours with greater efficiency than the unselected population [4]. Given the relevance of CSCs to tumourigenesis and metastasis [5], [6], more effective tumour therapies require a better knowledge of the characteristics of this subset of cancer cells and of the factors, extrinsic and intrinsic, which contribute to their ‘stemness’. Assessing the relevance and physiological role of the “stem cell markers” to the stem cell phenotype will substantially increase our understanding of CSCs and should aid in devising selective therapies.

In the case of colorectal cancer stem cells (CCSC) at present the best characterized “stem cell” markers are the surface antigens CD133 [4], [7] CD166 [8], CD44 and CD24 ([9],[10] (Reviewed by [11]). Intracellular markers of CCSCs include Musashi-1 ([12], [13]), Bmi-1 [14] and ALDH [15] (reviewed in [16]. However the most selective and promising marker of the stem cell in intestinal epithelium and of the intestinal cancer stem cells is LGR5 [17] (UNIPROT Accession # O75473; UNIGENE # Hs.658889; also called GPR49). In normal intestine LGR5 expression is restricted to the stem cell zone at the base of the crypt [18] and single cells from the small intestine expressing LGR5 can generate structures resembling intestinal crypts ‘in vitro’ [19], [20]. Most importantly, Barker et al. [21] have shown in mouse models that intestinal tumours arise from LGR5 positive cells, suggesting it marks the intestinal cancer stem cells. LGR5 is overexpressed in human colorectal adenomas and carcinomas relative to normal mucosa [22]: thus LGR5 overexpression is detected from the early stages of colorectal tumourigenesis. LGR5 is a wnt target gene [23], and the wnt pathway is activated early in the progression of the majority of colorectal cancers through truncations of APC (Adenomatous Polyposis Coli) and, less frequently, mutations of β-catenin (reviewed by [24]). It is unclear, however, whether LGR5 upregulation in colorectal cancer cells contributes significantly to tumourigenesis through maintenance of colorectal CSC, or is simply a reflection of activated wnt signalling, with no direct functional role.

Little is known about LGR5 function in development and carcinogenesis. LGR5 is an ‘orphan’ receptor belonging to the G-protein receptor coupled (GPCR) family [25]; its ligand and mode of intracellular signalling are at present unclear [26]. Knockout of LGR5 in mice results in neonatal mortality associated with craniofacial defects (ankyloglossia) [27]. A thorough study by Garcia et al [28] of prenatal intestinal development in GPR49-LacZ mutant mice (LGR5 null) shows that loss of LGR5 does not affect proliferation or migration of intestinal cells. However the authors noted a strong induction of Paneth cell differentiation in LGR5 knockout embryos, and a molecular signature characteristic of upregulated wnt signalling.

As LGR5 appears to be a marker of CCRCs, we have investigated which parameters of cell growth and differentiation are affected by modulation of LGR5 expression in colorectal cancer cell lines. Due to the functional redundancy of many signalling molecules and the strong feedback loops that maintain homeostasis, these studies are difficult to interpret in animal models, while low transfection efficiencies and restrictions on long-term culture prevent these studies in human primary tumour samples. To circumvent these difficulties we have used two colorectal carcinoma cell lines, LIM1215 [29] and LIM 1899 [30] as a model system. Our results show that LGR5 silencing and overexpression have opposing effects on cell phenotype, including anchorage-independent growth, migration and tumour formation as xenografts in mice. Paradoxically, suppression of LGR5 expression enhances tumourigenesis and is linked to a more mesenchymal phenotype. A study of the gene expression patterns after modulation of LGR5 cellular levels by siRNA knockdown or transgenic overexpression shows that loss of LGR5 upregulates wnt response genes and key EMT pathway genes; conversely, overexpression of LGR5 favours cell-cell adhesion. These results highlight the importance of LGR5, not simply as marker of colorectal tumour cells, but as a regulator of wnt responses, cell motility and cell-cell adhesion.

## Results

### LGR5 is expressed in colorectal cell lines with β-catenin mutations and upregulated by wnt stimulation in cells with APC mutations

**Figure 1:**
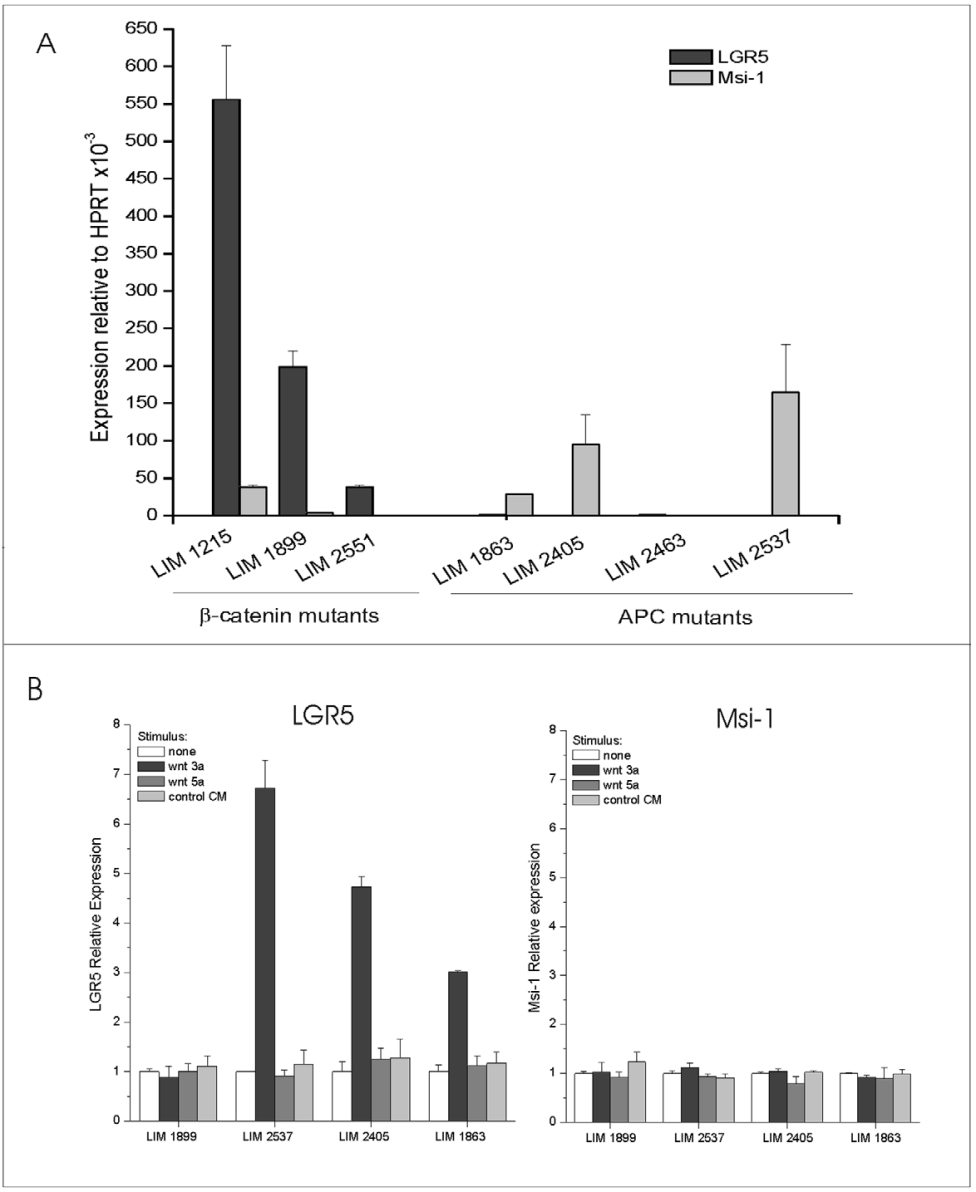
Expression of stem cell markers in CRC cell lines and selective induction of LGR5 by wnt 3a. A) Expression levels for LGR5 and Msi-1 were determined by qRT-PCR as described in Methods. Results are presented as gene expression relative to the endogenous control HPRT within each cell line. Cells have been grouped according to their β-catenin or APC mutational status. B) Cell lines carrying β-catenin mutation (LIM 1899) or APC mutations (LIM2537, LIM2405, LIM1863) were stimulated for 12 hrs with culture medium (no stimulus), with conditioned medium from L-cells expressing wnt 3a or wnt 5a, or with untransfected L-cell conditioned medium (control CM). Expression of LGR5 (left panel) and Musashi-1 (right panel) was determined by qRT-PCR as described in Methods. For each cell line, bars represent expression level of stimulated relative to unstimulated cells.

Colorectal tumours are characterized by mutations in wnt pathway signalling components [31],[32],[33], principally APC and β-catenin, leading to disregulated or cell-autonomous responses to wnt. LGR5 is overexpressed in primary colorectal tumours [34], [35]: overexpression could conceivably be due to enrichment of ‘stem-like’ cells, to upregulation of the wnt signalling pathway, and/or to wnt pathway-dependent maintenance of ‘stemness”. We utilized a panel of previously characterized human colorectal carcinoma cell lines [33] to compare LGR5 expression to the expression of another putative intestinal stem cell marker, Musashi-1 (Msi-1) [36],[12], [37]. Cells expressing high levels of LGR5 do not generally express high levels of Msi-1, and vice versa (Fig. 1A). Interestingly, we detected elevated levels of LGR5 mRNA only in cell lines carrying β-catenin mutations (Figure 1A). The elevation of LGR5 in β-catenin mutant cells is striking, suggesting that mutational activation of β-catenin is responsible for overexpression of LGR5, while mutation of APC is not sufficient to induce detectable LGR5 expression in these cell lines. To test the wnt dependence of LGR5 expression, we stimulated the cells with L-cell derived wnt3a, wnt5a or control conditioned media. LGR5 and Musashi-1 mRNA levels were tested in parallel by qRT-PCR 12 hrs after stimulation (Fig. 1B). As expected, LGR5 expression levels are unaltered by wnt3a stimulation in the β-catenin mutant cell line LIM1899 but selectively upregulated by wnt3a in APC-mutant cell lines LIM 2537, LIM 2405 and Lim1863 (Fig. 1B, left panel). Wnt stimulation did not affect the expression levels of Msi-1 in any of the cell lines tested (Fig. 1B, right panel). Upregulation of LGR5 by canonical wnt signalling in responsive cell lines was confirmed by immunostaining with a validated antibody to LGR5 (Fig. S1A). In these experiments, maximal levels of LGR5 protein were observed 48 hrs after stimulation of the cells with wnt3a, while neither wnt 5a or L-cell conditioned medium induced LGR5 expression (Fig. S1B and data not shown). Thus elevated levels of LGR5 in colorectal cancer cells are likely to be secondary to activated canonical wnt signalling, and mutations in β-catenin bypass the requirement for exogenous ligands. We have previously shown that LIM cell lines with heterozygous APC mutations have weakly activated wnt signalling resulting from autocrine production of canonical wnts [33]: the lack of LGR5 overexpression in these cells in the absence of exogenous wnt3a suggest a threshold effect for LGR5 induction.

### Modulation of LGR5 expression has profound effects on clonogenicity and tumourigenesis

**Figure 2:**
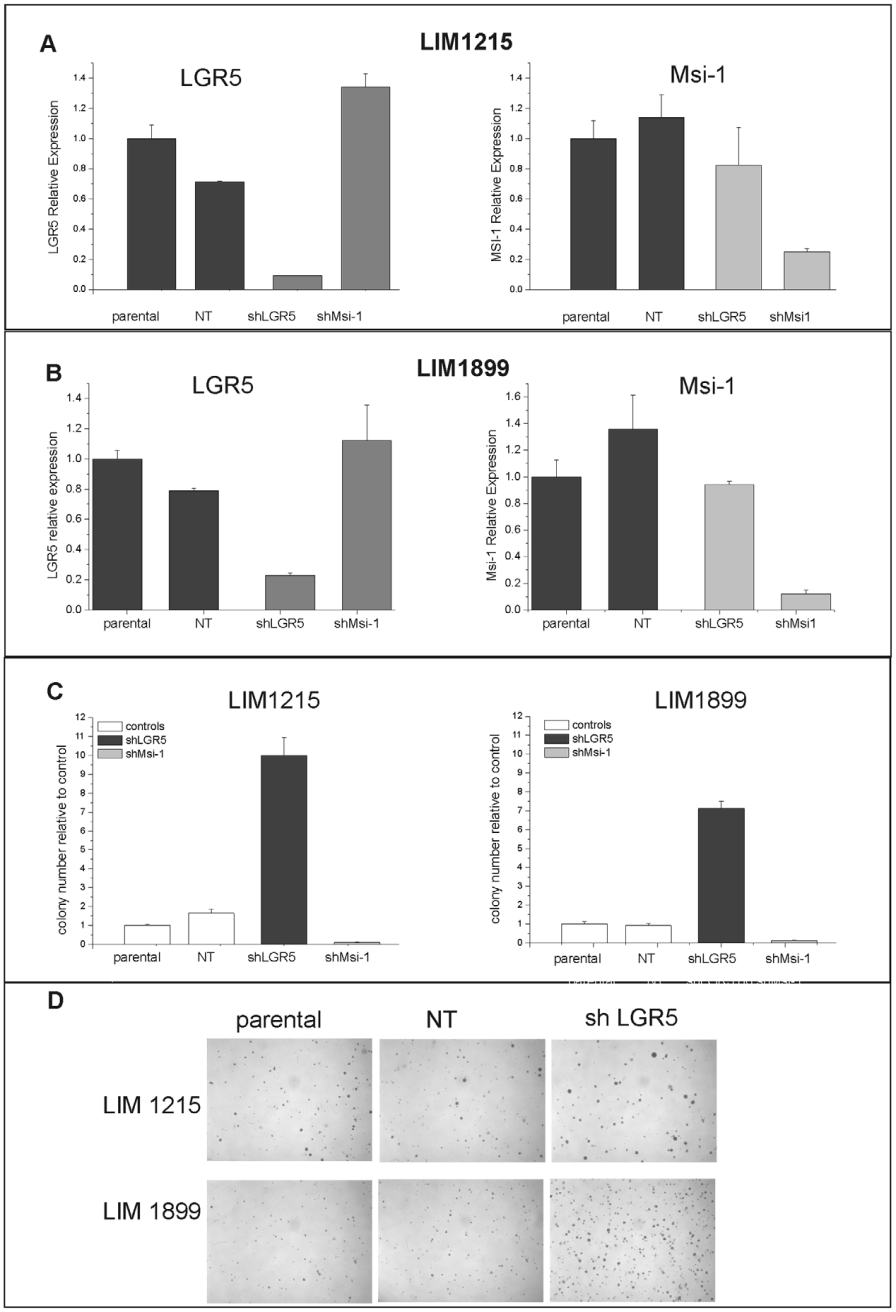
shRNA knockdown of LGR5 and Msi-1 have opposite effects on cell growth in soft agar. A) and B): LIM1215 (A) and LIM1899 (B) cells were transduced with lentiviral particles containing shRNA to non target sequences (NT), to LGR5 (shLGR5) or to Musashi-1 (shMsi-1) and bulk selected in puromycin. Expression of LGR5 (left panels) and Musashi-1 (right panels) was assessed by qRT-PCR two weeks after transduction. Data are presented as gene expression relative to the parental cell lines. These results are representative of >5 separate experiments. C): Cells expressing shRNAs (shLGR5, shMsi-1 and NT) and parental cells were grown in antibiotic-free medium for three days then tested for their ability to form colonies in soft agar as described in Methods.. Data are presented as colony forming efficiency of test samples relative to control (untransfected) parental cells and are the average and sd of three separate experiments. D): Representative images of colonies in soft-agar plates stained with crystal violet. Images were acquired with a Nikon 90i with a DXM 1200C camera.

If overexpression of LGR5 in colorectal cancer cells is mediated by hyper-activated wnt pathway, what role does LGR5 play in wnt responses, and does expression of LGR5 contribute to the maintenance of “cancer stemness”? To address the functional relevance of LGR5 expression in CRC cell lines, we reduced its expression in cells carrying a β-catenin mutation (LIM1215 and LIM1899) using inhibitory RNAs. We initially utilized lentiviral transduction of shRNA (short hairpin RNA) to LGR5. As controls, we used shRNAs directed to random sequences (non-target, NT) or to Msi-1. Musashi-1 is expressed in immature intestinal cells [12], [37] and is overexpressed in colorectal tumours [35], but is not a wnt-response gene (Fig. 1B). We used four separate shRNA constructs for each target gene: all were effective, and subsequent experiments were conducted using the most efficient shRNAs. Transduced cells were bulk selected in puromycin for two weeks to enrich for the shRNA-expressing cells, then switched to antibiotic-free media for functional characterization. Knockdown efficiency was monitored by qRT-PCR and cell proliferation was assayed using MTT assays and colony formation in soft agar. Lentiviral delivery of shRNA to LGR5 or to Musashi-1 was effective in both cell lines and lead to a marked and specific reduction in expression of the target genes (Fig. 2 A,B, B). The expression levels of the related genes LGR6 and Msi-2 were unaffected (data not shown). We confirmed loss of LGR5 protein after knockdown using immunofluorescence (Fig. S2), as LGR5 antibodies are not suitable for the detection of endogenous levels of this protein by Western Blot.

Knockdown of either LGR5 or Msi-1 levels did not affect the growth of cells as adherent monolayers (Fig. S3A), however loss of LGR5 and Msi-1 had striking and opposing effects on the clonogenicity of the cells in soft agar (Fig. 2C). Knockdown of Musashi-1 lead to a reduction in the colony forming ability of both LIM1215 and LIM1899 cells, consistent with the loss of proliferation and tumour forming ability of the colorectal cell line HCT116 after downregulation of Msi-1 as reported by Sureban et al [13]. In contrast, loss of LGR5 caused a reproducible and profound *increase* in the clonogenicity of both LIM1215 and LIM1899 (Fig. 2 C,D). These effects on colony formation were observed consistently in both cell lines and using two separate, LGR5-specific shRNA constructs.

Selection of the cells in puromycin might have led to changes in the expression of genes other than LGR5, contributing to this surprising result. We repeated the knockdown experiments using transient expression of Cy3-labelled siRNA (small hairpin RNA) to LGR5. Cells were transfected with the constructs and the expression of the Cy3-labelled siRNA was monitored by fluorescence microscopy. Transfection efficiency, assessed by Cy3 expression using fluorescence microscopy, was >80% in LIM1899, but <20% in LIM1215; consequently subsequent experiments were performed using LIM1899 cells.

**Figure 3:**
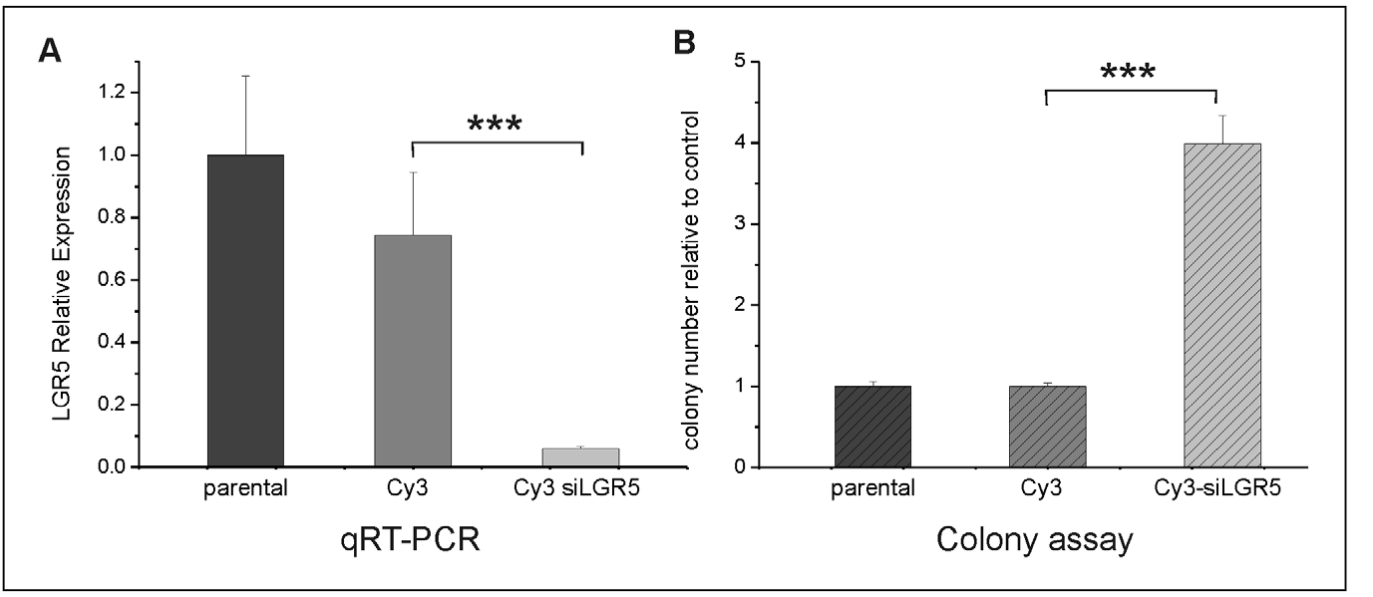
Knockdown of LGR5 by siRNA. LIM1899 cells were transfected with vector expressing Cy3 (Cy3) or Cy3-siRNA to LGR5 (Cy3 siLGR5). A) LGR5 expression by qRT-PCR in untransfected cells (parental) and cells transfected with Cy3 or Cy3-siRNA to LGR5. Plots represent LGR5 expression of test samples relative to the parental (untransfected) cell line. B) LIM 1899 cells were plated in soft-agar two days after transfection at 5×103 cells/ml and cultured for 10 days. Colony numbers were assessed after staining with crystal violet using a dissecting microscope. Plots represent colony number in test samples relative to the parental cells. The increase in colony numbers upon silencing of LGR5 is extremely significant (*** = p<0.0001 by the unpaired t-test). For both panels data are mean and sd of triplicate samples, and are representative of at least 3 separate experiments.

Knockdown of LGR5 by siRNA was very efficient (>90%) and specific (Fig. 3A). Importantly, shRNA and siRNA knockdown of LGR5 had identical effects on the clonogenicity of LIM1899, confirming that this phenotype is the result of LGR5 downregulation and is not an artefact of the selection process (Fig. 3B).

Since a decrease in LGR5 levels specifically enhances the clonogenicity of colorectal cancer cell lines, we investigated whether LGR5 overexpression would reduce the growth of these cells in soft-agar by performing both transient and stable overexpression of LGR5 in colorectal cell lines. We chose to use the same cell lines for both silencing and overexpression of LGR5 in order to minimize non-LGR5 specific changes in cellular parameters. While this strategy risks underestimating the effects of LGR5 overexpression, it allows a direct comparison between transfected cells and facilitates the interpretation of expression profiling.

**Figure 4:**
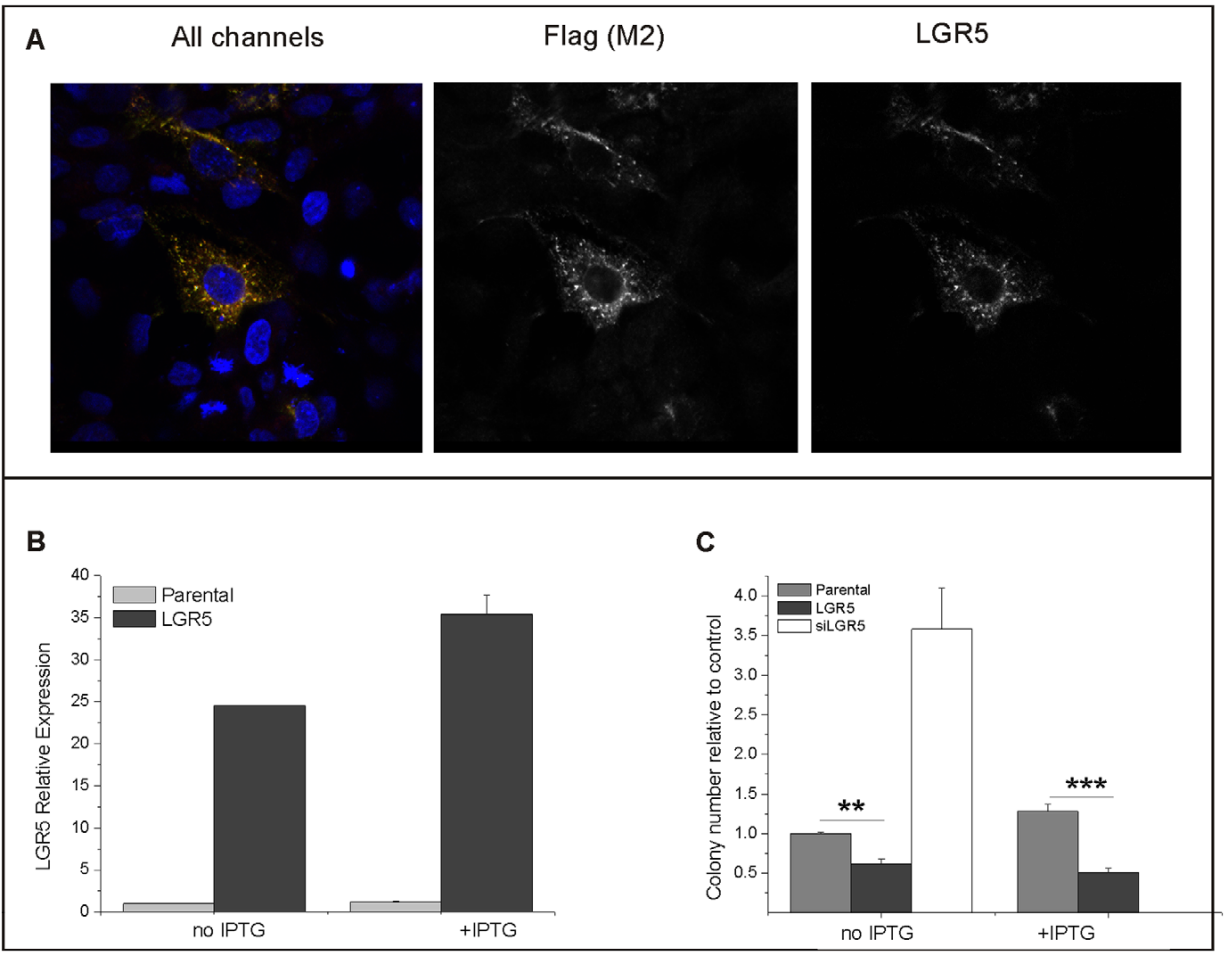
Overexpression of flag-tagged LGR5 and its effect on clonogenicity in LIM 1899 cells. LIM1899 cells were transfected with pTune vector containing human LGR5 flanked by a flag sequence and analysed 3 days after transfection. **A**) Confocal microscopy: LIM 1899 cells transfected with pTune/LGR5 were co-stained with anti-flag (M2) antibody followed by Alexa488 anti-mouse Ig (green), anti-LGR5 antibody HPA012530 followed by Alexa 546 anti-rabbit Ig (red) and the nuclear stain DAPI. Shown is a merged image (all channels) and greyscale images of the green and red channels, respectively. Confocal microscopy was performed as described in Methods. **B**) qRT-PCR: untransfected cells (parental) and cells transfected with the LGR5 expression vector were cultured for three days with or without IPTG (100 µM). RNA extraction and qRT-PCR were performed as described in Methods. The graph shows the level of expression of LGR5 in transfected relative to parental cells. Data are the mean +/−sd of triplicate samples, and are representative of >3 separate experiments. **C**) Clonogenic assay: untransfected cells and cells transfected with pTune/LGR5 or with Cy3-siRNA to LGR5 were seeded in soft-agar plates at 5×103 cells/plate as described in Methods. IPTG (100 µM). was added to triplicate plates for parental and LGR5-expressing cells only. Plates were incubated for 10 days then stained with crystal violet and colonies counted with a dissecting microscope. The graph shows means and standard deviation of three separate experiments, each normalized to the colony numbers for parental cells. The difference between parental and transfected cells is highly signficant (** = p<0.005 and *** = p<0.001 for the two culture conditions).

LIM1899 and LIM1215 cells were transfected with pTUNE vector containing the human LGR5 sequence flanked by myc and flag sequences. The efficiency of transfection for LIM1899 varied in these experiments between 30 and 60% as assessed by immunostaining of the cells with anti-flag antibodies, while transfection efficiency was between 10–20% for LIM1215 cells: hence subsequent experiments were carried out in LIM1899 cells. Overexpression of flag-tagged LGR5 in transfected cells was confirmed by qRT-PCR and by immunofluorescence using anti-flag and anti-LGR5 antibodies (Fig. 4 A,B). Over-expressed LGR5 was present both in the cytosol and at the plasma membrane; with accumulation in punctuate structures (Fig. 4A). This distribution is similar to the distribution of endogenous LGR5 in colorectal cancer cells after wnt stimulation (Fig. S2). The pTune system is designed for IPTG- inducible expression of proteins, however high levels of expression were present in the absence of IPTG, with only a moderate increase after induction (Fig. 4 B). Overexpression of LGR5 in LIM1899 resulted in a significant loss of colony-forming ability in soft agar (Fig. 4C) without affecting proliferation under adherent conditions (Fig. S3). Parallel transfection of the cells with Cy3-siRNA to LGR5 caused the expected increase in colony numbers in the same assay (Fig. 4C). Stable cell lines overexpressing LGR5 were generated by selection of the transfected cells in neomycin: LGR5 expression in these cell lines, as assessed by qRT-PCR and immunofluorescence, was increased significantly (Fig. S4 A, B), and was inversely correlated with clonogenicity in soft agar (Fig. S4 C,D). Thus LGR5 modulation has consistent and specific effects on the clonogenicity of colorectal cancer cell lines.

**Figure 5:**
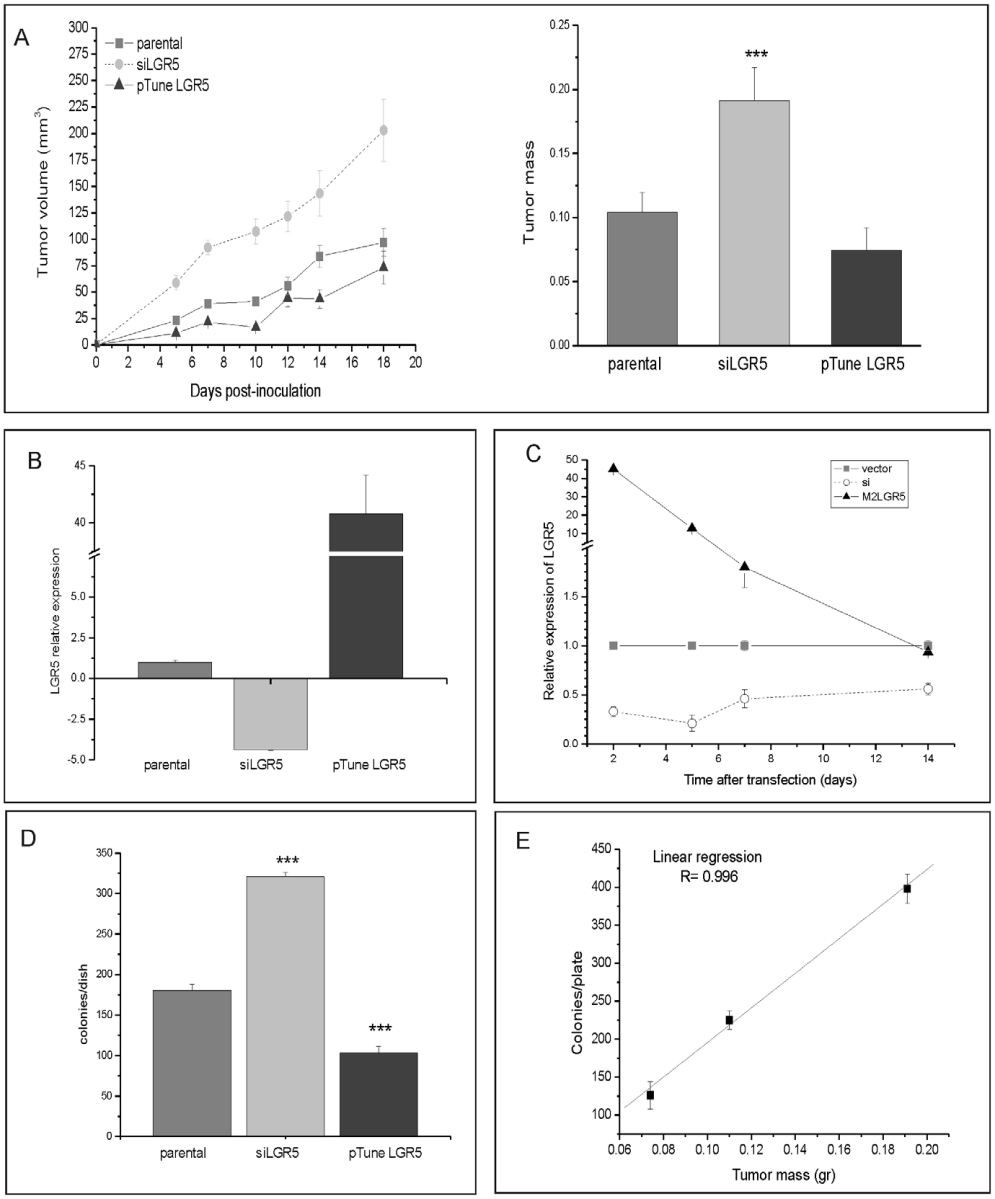
Xenograft tumour growth is affected by the levels of LGR5. LIM1899 cells were mock transfected (no vector), transfected with Cy3LGR5 or with pTune/LGR5. Cells were expanded for two doublings (48 hrs) after transfection, then collected for inoculation in nude mice, determination of growth in soft agar and measurement of LGR5 expression by qRT-PCR as described in Methods. **A**) Left panel: xenografts tumour growth curves, right panel: tumour mass at day18. The growth of subcutaneous tumours was measured three times weekly using callipers, and volume determined by the formula V = 1/2 (length×width^2^). At the end of the experiment (day 18) tumours were dissected and weighed. Data are averages and standard errors for each group (16 tumours/group). There was no statistical difference between parental and pTune Lgr5 tumour mass, however the difference between parental and siLGR5 tumours is significant (*** = p<0.001). B) A sample of cells used for xenograft injection was tested by qRT-PCR for LGR5 expression. Data were analysed in ABI 7300 (ΔΔCt study), and are presented as LGR5 expression relative to the parental cells. Data are presented as means and standard errors. C) Time course of transgene expression in cultured LIM 1899. The expression of LGR5 in cells transfected with siLGR5 or pTune LGR5 was followed over a period of two weeks by qRT-PCR. Levels of LGR5 expression are presented relative to vector control for each of the time points. D) A sample of cells used for the xenograft experiment was cultured in soft-agar plates at 5×10^3^ cells/plate to determine cloning efficiency. Plates were incubated for 10 days, then stained with crystal violet and colony numbers determined by light microscopy. *** = p<0.001. E) Correlation between tumour mass (graph A) and cloning efficiency (graph D).

Clonogenicity in semi-solid media of tumour cells often correlates with their ability to form tumours in immunocompromised mice. We used a xenograft model to test whether modulation of LGR5 affects the tumourigenicity of LIM1899 cells. LIM1215 was not tested in this system as it is both poorly tumourigenic as a xenograft and transfects with very low efficiency. LIM1899 cells were expanded and transfected in bulk with Cy3-siRNA to LGR5 or with pTune-LGR5. Transfected and parental cells were expanded for two days, then harvested for parallel determination of LGR5 expression by qRT-PCR, clonogenicity ‘in vitro’ and tumour-forming capacity ‘in vivo’ (Fig. 5). The proportion of transfected cells, monitored by fluorescence microscopy for Cy3siRNA expression and immunostaining with anti-flag antibody for LGR5 overexpression, were 80% and 60%, respectively. The cells used for the xenografts had the expected reduction or increase in LGR5mRNA (Fig. 5B): suppression of LGR5 expression persisted for up to two weeks in cells treated with siRNA, while overexpression of LGR5 had returned to baseline by 14 days (Fig. 5C). The clonogenicity of the cells used in xenografts was inversely proportional to the level of expression of LGR5 (Fig. 5D). Cells expressing siRNA to LGR5 showed enhanced tumour formation; conversely, cells overexpressing LGR5 were less tumourigenic (Fig. 5A,B). The difference in tumour size between the LGR5 knockdown and the parental cells was highly significant (p<0.0001) at all time points, however the growth of tumours overexpressing LGR5 differed significantly from parental cells only for the first 10 days of the xenografts experiment (Fig. 5A). The difference in the stability of expression of the siRNA to LGR5 vs. the LGR5 construct (Fig. 5C) is consistent with the long-term effects of LGR5 downregulation and the more transient effects of LGR5 upregulation on the xenografts. We observed very good correlation (R = 0.996) between clonogenicity in soft agar and tumourigenicity (Fig. 5D), confirming the validity of the former as a substitute assay.

**Figure 6:**
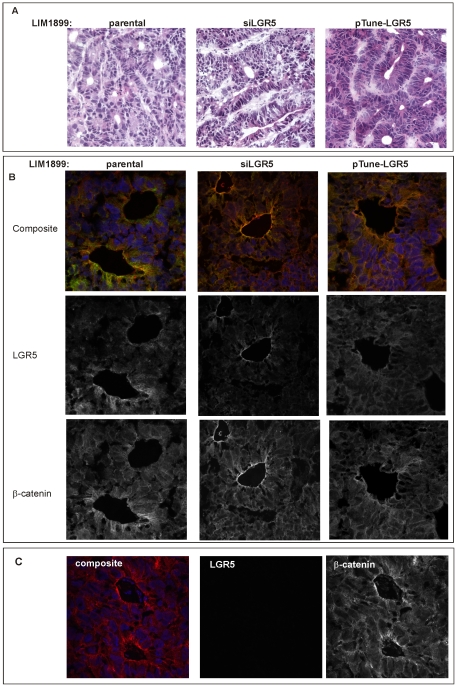
Morphology and LGR5 reactivity of xenografts tumours. A) Haematoxilin and eosin staining of frozen sections from representative tumours generated from parental LIM1899, LIM1899 transfected with siRNA to LGR5 or LIM1899 transfected with pTune-LGR5 construct. Brightfield images were acquired on a Nikon 90i microscope with a 20× lens. B) Confocal images of frozen sections stained with antibodies to β-catenin (red), LGR5 (green) or the DNA stain DAPI (blue). All images are Z-stacks of confocal sections. For each set, the upper panel shows the three combined stains, the middle panel LGR5 (greyscale), and the bottom panel β-catenin (greyscale). Images were obtained on a Nikon C1 confocal microscope with a 60× oil lens. C) Specificity control for LGR5 staining: frozen sections were stained with antibody to β-catenin and normal rabbit serum, followed by Alexa 488 anti-rabbit Ig (green) and Alexa 546 anti-mouse Ig (red) and the nuclear stain DAPI (blue). The green channel (NRS) and red channel (β-catenin) are shown separately in greyscale. Images were obtained as in B).

We analysed the subcutaneous tumours for LGR5 expression using immunofluorescence. Representative images of the tumours stained with heamatoxilin and eosin (A), or co-stained with LGR5 and β-catenin (immunofluorescence images) are shown in Fig. 6. Morphologically, all tumours consisted of well defined glands, and could be classified as moderately differentiated adenocarcinomas (Fig. 6A). Tumours derived from siLGR5 LIM1899 tended to have a more disordered morphology, reflected in the subtle but significant difference in the number of well-formed glands per field between parental tumours (26+/−3 n = 16) and siLGR5 tumours (15+/−2 n = 16; p = 0.0014). The number of glands per field was increased in tumours overexpressing LGR5 (31+/−2, n = 16) however the difference from the parental tumours was not statistically significant. In all tumour samples LGR5 staining was most prominent in the glands, particularly towards the gland lumen, and was weaker in the more amorphous areas of the tumour (Fig. 6B). Staining for LGR5 was specific, as no signal was detected in samples incubated with normal rabbit serum (Fig. 6C). LGR5 immunoreactivity was marginally elevated in tumours derived from LIM1899 cells transfected with pTune LGR5, but not in all areas of the tumours. LGR5 staining was decreased, but not totally absent, in tumours derived from LIM1899 cells transfected with siLGR5. In these samples, LGR5 expression appeared restricted to the glandular structures (Fig. 6B).

### Cell-cell adhesion and migration are regulated by LGR5 levels

LGR5 levels have profound effects on the anchorage independent proliferation of colorectal cancer cells ‘in vitro’ and ‘in vivo’; however how LGR5 modulates anchorage-independent growth is unclear. We observed that LIM1899 cells overexpressing LGR5 tend to grow in ‘colonies’ with tight cell-to-cell contacts, while LIM1215 and LIM1899 cells with reduced LGR5 are more diffuse on the plastic surface (not shown). The opposite phenotypes observed after knockdown or overexpression of LGR5, and their consistency in transient and stable expression systems, indicate that this phenomenon is directly correlated to LGR5 levels. Thus LGR5 may modulate the balance between cell-cell and cell-substrate adhesion. To characterize cell-matrix and cell-cell interactions we cultured cells as spheroids in hanging drops [38] and performed both wound assays and motility assays to measure the migration potential of the cells. Hanging drops assays measure the proliferative potential of the cells in the absence of cell-matrix interactions; wound assays assess the rate of movement of a cell monolayer, and the motility assay measures the rate of migration of the cells through the ECM in filter pores.

**Figure 7:**
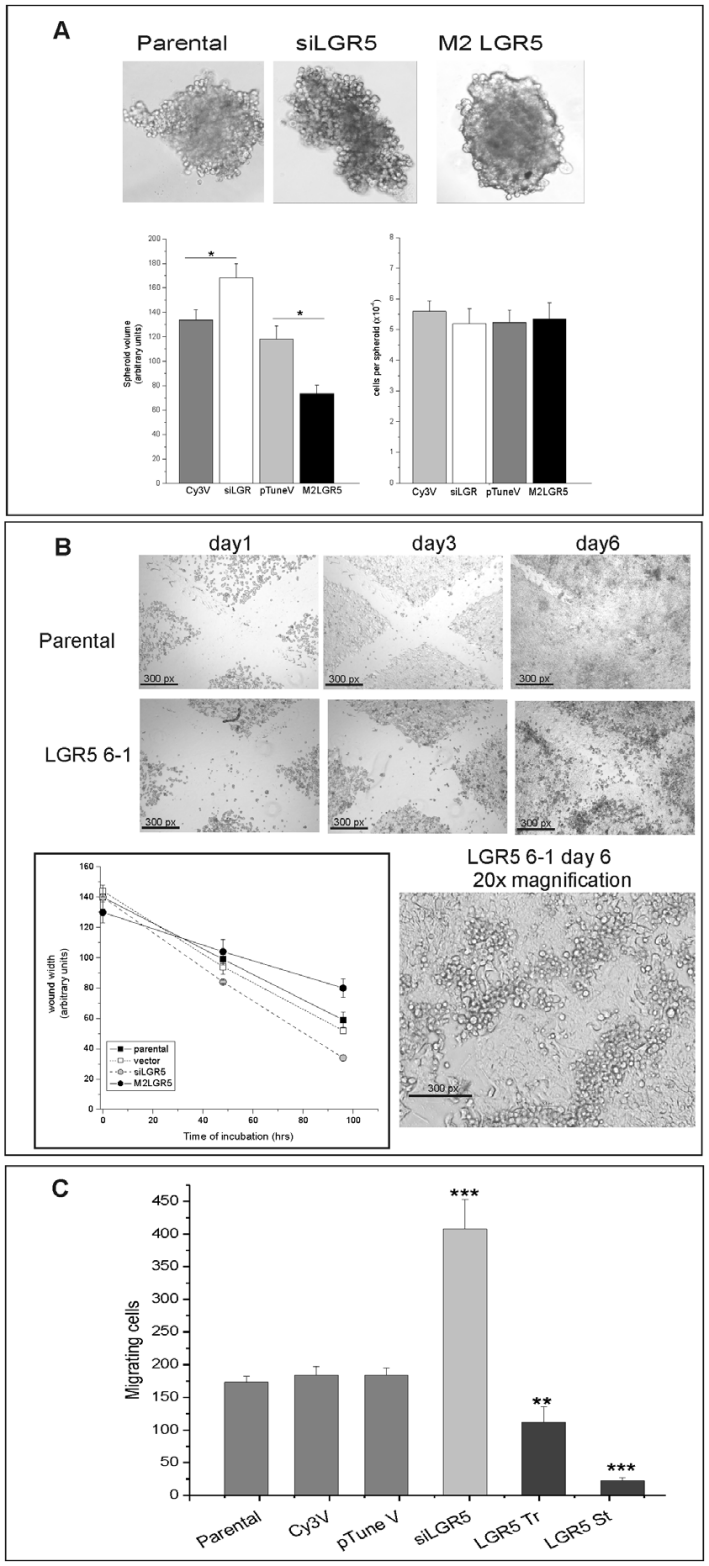
Effects of LGR5 modulation on cell-cell adhesion and migration. Lim1899 cells were transfected with vector controls (Cy3V and pTuneV), with Cy3- siLGR5 or with pTune/LGR5. Parental, transiently transfected cells (Tr) or stably transfected cell lines (St) were cultured under the following conditions: A) Cells were seeded in 30 µl droplets on a plastic surface, and the plate inverted to create hanging drops as described in Methods. Images were taken after 8 days by re-inverting the plastic support and imaging in bright field with a Nikon 90i microscope and a 10× lens. Digital images were acquired with a Photometrics CoolSnap digital camera. Spheroid volumes (left-hand graph) were calculated from these images using the modified ellipsoid formula. Spheroid sizes differed significantly between siLGR5 or M2LGR5 transfected cells and their counterparts transfected with empty vector (p = 0.0312 and p = 0.321, respectively, by the unpaired t-test). The cellularity of the spheroids (right hand graph) was assessed as described in Methods. In both graphs data represent the mean and standard deviation of 10 individual spheroids per cell line. B) Parental cells and cells stably transfected with pTune/LGR5 (clones 6-1) were plated at high density in 24-well plates. Wounds were scratched in the adherent monolyers and the wells were imaged every two days with a Nikon90i microscope using a 10× lens (upper panels). The photomicrograph on lower right shows a higher magnification of LGR5 6-1 wound at day 6 (20× lens). Insert: Rate of wound closure over 96 hr. C) Cells were seeded in Transwell inserts (8 mm pore size) and cultured for 4 days. Filters were fixed and stained with May-Grumwald/Giemsa. Cells on the upper side of the filters were removed, and filters mounted on glass slides. Cells present on the underside of the filters (migrating cells) were counted by light microscopy as described in Methods. The graph presents average and sd of three separate samples for each cell type. Tr and St denote transient and stable LGR5 transfectants. Significance levels were determined by the unpaired t-test. *** = p<0.001; ** = p<0.005.

Parental LIM1899 cells, or LIM1899 cells transfected with empty vectors, grow in hanging drops as aggregates with dense centres (Fig. 7A). In parallel cultures, spheroids of cells with reduced levels of LGR5 (siLGR5) are surrounded by a halo of loosely-associated cells and are easily disrupted, while cells expressing high levels of LGR5 (M2LGR5), either transiently or stably, pack into compact spheroids resistant to mechanical disruption (Fig. 7A and data not shown). The difference in cell density is reflected in the volume of the spheroids relative to their cellularity: while spheroids from different cell lines contain similar number of cells (Fig. 7A, right hand graph), the difference in volume between siLGR5 spheroids and spheroids overexpressing LGR5 (Fig. 7A, left hand graph) is significant (p = 0.001) reflecting a tighter packing of the cells in M2LGR5.

In wound repair assays LGR5 overexpressing cells have a reduced ability to repopulate the scratch wound compared to the parental cells (Fig. 7B), and accumulate at the edge of the wound forming a densely packed ridge of viable cells (Fig. 7B, larger magnification). Both assays confirm the original observation that LGR5 overexpression favours cell-to-cell adhesion. Transwell assays were used to monitor the migration ability of cells with different levels of LGR5. Cells were seeded in Transwell filters and incubated for three days, before switching the upper filter contents to serum-free medium to encourage migration. Under these conditions, cells with reduced levels of LGR5 migrated to the underside of the filters to a much greater extent than untransfected cells, while cells overexpressing LGR5 had significantly reduced migration (Fig. 7C). These differences in migrating cell numbers were highly significant: p = 0.002 for siLGR5, p = 0.03 and p = 0.001 for transient (LGR5-Tr) and stable (LGR5-St) overexpressors, respectively. Consistently, the reduction in motility displayed by LGR5 overexpressing cells was proportional to the level of LGR5 expression.

**Figure 8:**
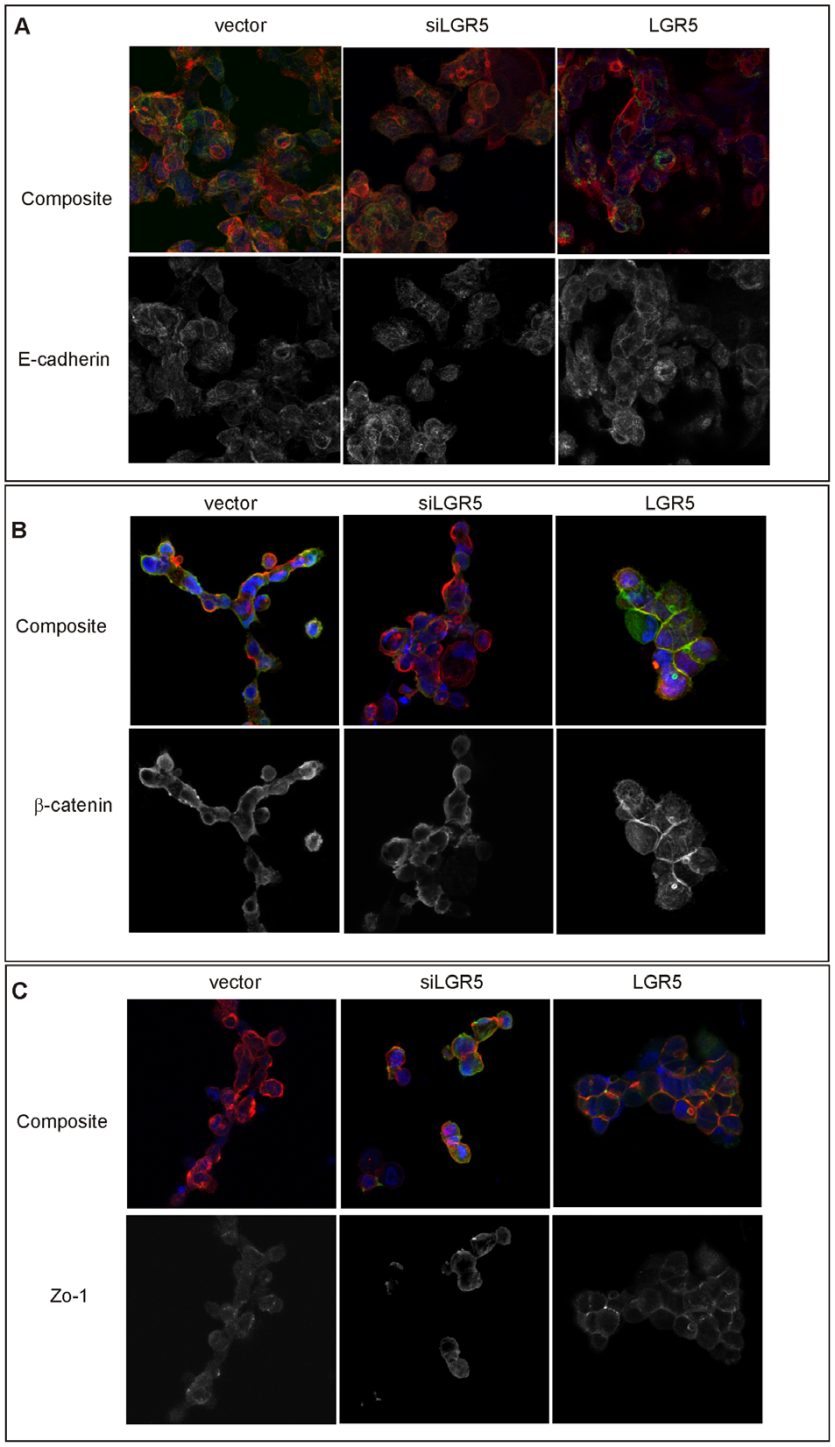
Expression of junctional proteins in Lim 1899 cells with altered LGR5 expression. Cells transfected with empty vector, siRNA to LGR5 (siLGR5) or pTune/LGR5 (LGR5) were grown in chamber slides and prepared for immunofluorescence as described in Methods. Slides were stained with antibodies to E-cadherin (**A**), β-catenin (B) or Zo-1 (C) followed by Alexa 488 anti-mouse Ig (green channel in all samples). Cells were counterstained with rhodamin-phalloidin (red channel) and the nuclear stain DAPI (blue channel). Images are Z-stacks of sequential confocal images. For each antibody, the top panel shows the merged channels and the bottom panel the green channel only (grey scale).

To understand the changes in adhesion and motility, we used confocal fluorescence microscopy to monitor the localization and expression of the adherens junction proteins E-cadherin, β-catenin and Zo-1 in matched samples of parental, LGR5 knockdown or LGR5 overexpressing LIM1899 cells (Fig. 8). Fixed cells were incubated with the appropriate antibodies and fluorescent secondary antibodies, and co-stained with rhodamine-phalloidin to visualize actin. Neither the levels nor distribution of E-cadherin changed significantly with up-or down-regulation of LGR5 (Fig. 8A), however there was a consistent recruitment of β-catenin to the cell junctions in LIM1899 overexpressing LGR5 (Fig. 8B). This was surprising as the LIM1899 cell line carries an activating β-catenin mutation resulting in a predominantly cytosolic β-catenin, with some weak association to the membranes but rarely seen at the cell-cell junctions; this distribution is insensitive to wnt signalling stimulation or inhibition [33]. The tight-junction molecule Zo-1 was also increased at the cell-cell contacts in LIM1899 cells overexpressing LGR5 (Fig. 8C). The relative amounts of these proteins in cells under- or over-expressing LGR5 were also assessed by immunoblotting (Fig. S5 A, B), confirming little change in total β-catenin levels, marginal increase in E-cadherin (albeit not statistically significant), and significant increase of Zo-1 (Fig. S5B). Overall, these results are consistent with an enhancement of cell-cell adhesion in cells overexpressing LGR5.

**Figure 9:**
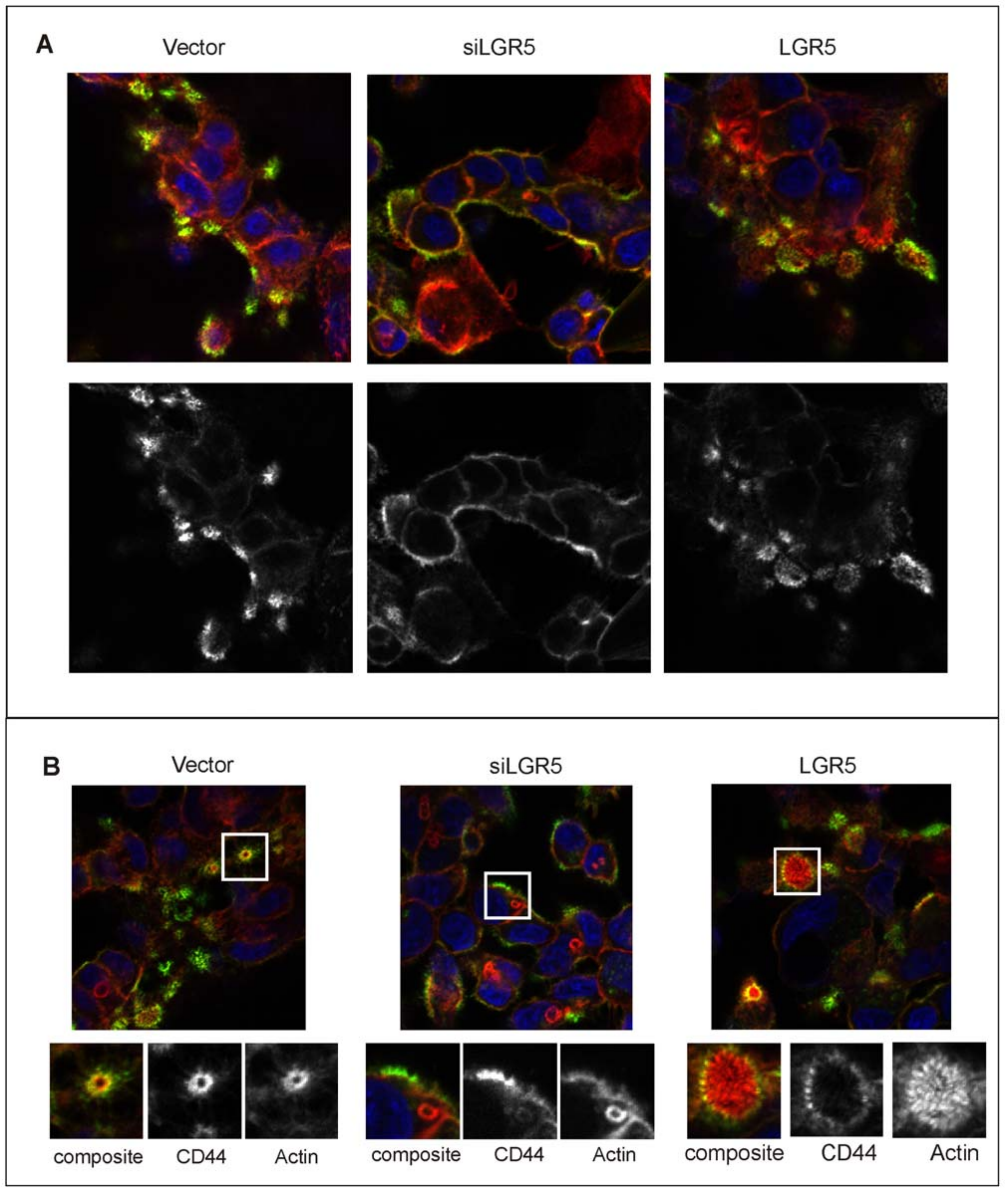
Distribution of CD44 in cells with altered levels of LGR5. Lim 1899 cells expressing siRNA to LGR5 (siLGR5) or the pTune/LGR5 construct (LGR5) were seeded on chamber slides, fixed and stained with rhodamine-phalloidin (red) or anti-CD44 followed by Alexa 488 anti-rat (green). Nuclei were stained with DAPI (blue). A): typical patterns of CD44 localization in the different cells. B) micrographs selected to show the focal actin rings and their coincidence (vector, LGR5) or lack of coincidence (siLGR5) with CD44. The lower panels show enlarged areas of the micrograph, highlighting the actin structures associated with CD44. In these panels actin staining and CD44 staining are shown separately in greyscale.

Given the effects of LGR5 modulation on cell migration, we hypothesized that LGR5 levels might affect, directly or indirectly, the expression or localization of adhesion molecules. CD44, CD133 and CD166 are adhesion molecules expressed on intestinal stem cells and colorectal cancer stem cells [8], [39], [40] and therefore can be expected to have overlapping expression patterns to LGR5. As these molecules have been used extensively as stem cell markers, we also wanted to assess whether changes in LGR5 expression resulted in altered patterns of expression for these markers. CD133 is not expressed on LIM1899, as assessed by flow cytometry (Fig. S6 A, B), while CD44 and CD166 are expressed at high levels. Only CD44 surface expression is weakly enhanced by upregulation of LGR5 in these cells (Fig. S6 B), but there is no appreciable change in total cellular CD44 as assessed by immunoblotting (Fig. S5). Confocal microscopy revealed that the cell surface distribution of CD44 is subtly altered in LGR5 knockdown cells. In parental LIM1899 cells and in cells overexpressing LGR5, CD44 associates with actin rings as assessed by morphology and colocalization with actin (Fig. 9 A,B). When LGR5 expression is reduced or abolished by inhibitory RNAs, CD44 is more uniformly distributed on the cell surface and is missing selectively from the focal actin rings (Fig. 9 A,B). This phenomenon was observed consistently with knockdown of LGR5, either by siRNA or shRNA, in both Lim1899 and LIM1215 cells (Fig. S7). The distribution of CD44 in LIM 1899 cells overexpressing LGR5 was unaltered. The F-actin circular structures we observe closely resemble the CD44-rich podosome rosettes described in the literature [41], [42]. These structures contain MMPs and are sites of collagen-directed matrix degradation [43]. It is likely that the loss of CD44 reactivity from these sites reflect localized CD-44 shedding, which is dependent on MMPs and promotes cancer cell migration and invasion [44]. While a detailed analysis of these ‘podosome-like” structures is beyond the scope of this paper, we have evidence of increased expression of the mRNA for EMT molecules, such as collagen and MMPs, when the levels of LGR5 are reduced (see next section). These results are consistent with the hypothesis that collagen and MMP-mediated CD44 shedding is responsible for the observed selective loss of CD44 reactivity from F-actin-rich structures when LGR5 levels decrease.

### Expression profiling of cells with altered LGR5 levels

There is no known role for LGR5 in any well-defined biochemical pathway or biological process, impeding a direct assessment of changes driven by altered LGR5 levels. To assess the pathways affected by LGR5 we performed expression profiling using pathway-directed arrays. Given the important role of LGR5-expressing cells in the intestine, we concentrated on three pathways which are of paramount importance for intestinal homeostasis and carcinogenesis, wnt, notch and EMT, using pathway-specific PCR arrays. Expression patterns of pathway-specific genes in LIM1899 and LIM 1215 cells after knockdown of LGR5 by RNAi, and in LIM 1899 cells after overexpression of LGR5, were compared to those of parental cell lines either untransfected or transfected with empty vectors. To maximize the robustness of the analysis, even at the cost of significance levels, we analysed both stable and transient transfectants as replicates.

Altered gene expression was considered specific to LGR5 modulation only if up-or down-regulation was higher than 2-fold relative to the control cells and these changes showed opposite trends in the LGR5 knockdown and LGR5 overexpressing cells. Genes which changed in similar way in both sets were discarded from the analysis. Tables 1, 2 and 3 list, in alphabetical order, the genes specifically affected by LGR5 modulation in the Wnt, Notch and EMT pathways, respectively. Many genes were differentially expressed between control and LGR5 up- **or** down-regulated cells, but were not significantly altered in the other experimental set: these genes are not included in Tables 1, 2, 3. Our analysis may thus be biased against genes already maximally regulated by the endogenous LGR5 levels. Tables 1, 2, 3 report the results for the LIM1899 cell lines, since we had both knockdown and overexpression samples for this cell line; the genes affected similarly by LGR5 knockdown in LIM1215 are indicated by asterisks. A full report of the analysis can be viewed in Tables S1, S2, S3, S4, S5, S6 and Figures S8, S9, S10. Overall, the wnt pathway analysis (Table 1) strongly supports the concept of an increase in canonical wnt signalling upon knockdown of LGR5: WISP1, Wnt5a, Fzd 7 and FGF4 have been reported to be upregulated following wnt3a stimulation in epithelial cells [45], [46] [47], [48], while Lef-1 [49] TCF7, TLE2 have been shown to be downregulated in response to canonical wnt signalling. Furthermore, the β-catenin/Tcf inhibitors CtBP and CTNNBIP1 [50] are downregulated after LGR5 silencing and upregulated upon LGR5 overexpression. The expression of only a few genes in the Notch pathway was altered after modulation of LGR5 (Table 2), and most are not restricted to the Notch signalling pathway (Fos, FosL1, Keratin1 and MMP7) or are of unknown significance in epithelial cells (e.g. SH2D1A and PTCRA). Notch2 and Notch2NL are massively downregulated after LGR5 silencing, albeit with low significance value (Table 2). As these genes are not upregulated with LGR5 overexpression in lIM1899 cells, the relevance of these findings is unclear. EMT genes are by far the most affected by LGR5 overexpression or knockdown, with selectively altered expression in 28 of the 84 genes in the array (Table 3). Suppression of LGR5 leads to strong upregulation of mesenchymal genes and of genes positively regulating EMT; these same genes are markedly downregulated upon LGR5 overexpression (note however that most of these changes have p>0.1). Particularly striking is the enhanced expression of extracellular matrix proteins and of matrix-metalloproteases. It must be stressed, however, that E-cadherin is not reduced and Vimentin is not upregulated upon knockdown of LGR5; hence the picture that emerges is of partial induction of EMT by loss of LGR5, which correlates very well with the morphological and functional observations described above (Fig. 7 and 8). This EMT-like program could either be directly mediated by LGR5, or result from enhanced wnt signalling, as many of the genes upregulated by LGR5 silencing and linked to EMT are also upregulated following canonical wnt activation [51], [52] [53], [54], [55], [56], [57]. These wnt responsive genes have been marked by a bullet in Tables 2 and 3. Wnt5a, which is significantly overexpressed after knockdown of LGR5, is also strongly linked to EMT: [58], [59] and may contribute significantly to the observed phenotypes.

**Table 1:** Changes in wnt pathway gene expression upon modulation of LGR5 in LIM1899 cells.

Gene	Fold changeLGR5 silencing	p value	Fold changeLGR5 overexpression	p value
WISP1*	**8.74**	0.03	−1.60	0.57
Wnt 5a*	**7.38**	0.004	**−3.53**	0.04
Casein Kinase 1γ*	**4.05**	0.04	−1.21	0.84
FGF4*	**3.41**	NA	**−6.33**	NA
Axin 1	**2.26**	0.07	−2.43	0.10
Frizzled 7*	**2.02**	0.004	**−2.55**	0.39
Lef -1*	**−4.39**	0.03	1.76	0.05
FRAT 1	**−3.20**	0.03	**2.28**	0.13
DIXDC1	**−2.82**	0.11	**3.14**	0.016
DAAM 1	**−2.52**	0.11	**2.00**	0.19
TLE2*	**−2.38**	0.02	1.89	0.21
Tcf 7 *	**−2.25**	0.25	**2.01**	0.18
CTNNBIP1	**−2.16**	0.05	**3.29**	0.01
Kremen 1*	**−2.24**	0.02	1.55	0.15
CtBP1	**−2.05**	0.04	1.45	0.15
CtBP2 *	**−2.52**	0.05	**2.16**	0.08

Expression levels of wnt pathway genes were analysed in three independent samples for each of parental cells, cells with LGR knockdown and cells with LGR5 overexpression using SABioscience pathway arrays. Data are presented as fold-change relative to the parental cells. Data were analysed as described in Methods using the SABioscience analysis program. Only genes with at least 2-fold change in either sample set, and for which there are significant differences between the two sample sets, are reported.

*the same trend in gene expression was observed in LIM1215 after knockdown of LGR5.

**Table 2:** Changes in Notch pathway gene expression with modulation of LGR5 in LIM1899 cells.

Gene	Fold changeLGR5 silencing	p value	Fold changeLGR5 overexpression	p value
WISP 1	**6.01**	0.07	**−2.78**	0.94
SH2D1a	**5.94**	0.004	−1.90	0.83
•MMP7	**5.35**	0.10	**−2.15**	0.35
•Keratin 1	**4.93**	0.58	**−3.95**	0.31
PTCRa	**4.73**	0.52	**−2.14**	0.34
Fos	**2.78**	0.94	−3.03	0.038
Notch 2	**−21.8**	0.13	1.27	0.91
Notch2NL	**−15.74**	0.20	1.55	0.76

•denotes genes reported in the literature to be affected by activation of canonical wnt signalling.

**Table 3:** Changes in EMT pathway gene expression upon modulation of LGR5 in LIM1899 cells.

Gene/Protein	Fold changeLGR5 silencing	p value	Fold changeLGR5 overexpression	p value
Collagen 1a	**6.721**	0.17	**−5.03**	0.29
Collagen 3a	**6.59**	0.16	−1.64	0.29
TFPI2	**13.1**	.14	**−2.60**	0.56
SPP1	**12.59**	0.13	**−2.28**	0.6
MITF	**10.43**	0.13	**−1.65**	0.72
FOXC2	**10.38**	0.07	**−2.85**	0.45
ESR 1	**10.18**	0.16	−1.91	0.64
N-cadherin	**9.50**	0.16	**−2.64**	0.59
•Fibronectin 1	**7.38**	0.16	−1.56	0.74
ZEB 1	**5**	0.10	**−3.45**	0.16
ZEB 2	**7.17**	0.10	**−2.54**	0.46
GNG 11	**6.8**	0.24	**−2.65**	0.58
•MMP2	**3.66**	0.09	**−5.67**	0.16
•MMP3	**6.43**	0.02	**−5.09**	0.12
•MMP9	**3.40**	0.03	**−7.91**	0.04
•KRT 14	**6.45**	0.13	**−4.33**	0.17
•KRT 7	**3.46**	0.08	−1.31	0.79
•SNAI 2	**5.31**	0.08	−1.13	0.09
•SNAI 3	**2.13**	0.30	−1.38	0.66
PDGFRb	**5.30**	0.11	**−2.84**	0.33
GSC	**5.03**	0.27	−1.37	0.85
MAP1b	**4.63**	0.20	**−3.63**	0.40
Caldesmon 1	**4.32**	0.16	**−2.81**	0.22
•Twist-1	**4.25**	0.28	**−4.97**	0.28
SPARC	**4.11**	0.09	**−7.02**	0.02
SOX 10	**2.43**	0.09	**−3.74**	0.67
STEAP 1	2.43	0.14	−1.75	0.3
•Versican	**2.31**	0.45	**−4.14**	0.30

•denotes genes which are reported in the literature to be upregulated following activation of canonical wnt signalling.

## Discussion

The data presented here support the view that LGR5 is a wnt response gene and its expression is induced and/or maintained during colorectal carcinogenesis [34],[26], [60]. However our investigation of the functional significance of LGR5 expression in the context of colorectal cancer cell lines show that, rather than contributing to the tumour phenotype, LGR5 antagonizes many of the accepted characteristics of tumour cells such as anchorage-independent growth, loss of cell-cell adhesion, enhanced migration and a switch from the epithelial to a more mesenchymal phenotype. In this cellular context, our observations that LGR5 suppresses responses to wnt signalling and antagonizes EMT are unexpected and appear paradoxical, but have significant implications for intestinal cell biology. While LGR5 may well mark the colorectal cancer stem cells, our data suggest that LGR5 is not functionally required for tumour expansion but may instead play a role in stem cell localization and restriction to a self-renewing niche.

Our results are consistent with a model where expression of LGR5 occurs with sustained levels of activation of the canonical wnt pathway. High expression levels of LGR5 occur only in cells with β-catenin mutations, not in cell lines with heterozygous APC mutation, unless exposed to extracellular wnt. While there is evidence of autocrine wnt signalling in these APC mutant cell lines [33] it may be insufficient to upregulate LGR5, or other factors may inhibit the upregulation of LGR5. In murine intestine, LGR5 expression is restricted to the base of the crypt, coincident with wnt signalling from the adjacent Paneth cells [20]. Interestingly, in neonatal mice loss of LGR5 in this compartment leads to premature differentiation of Paneth cells, suggesting reciprocal control between LGR5 positive stem cells and Paneth cells [28]. The maintenance of stemness, as opposed to differentiation, appears to be dependent on sustained wnt stimulation and on expression of LGR5. LGR5+ cells co-cultured with Paneth cells form organoids with crypt-villus architecture, but exposure of LGR5+ cells to exogenous wnt, or loss of APC, causes loss of differentiation and acquisition of a proliferative progenitor phenotype [20]: thus the ‘right’ amount of wnt signalling is required for differentiation. Barker et al, [21] have elegantly shown that, upon truncation of APC, cancer arises solely from the LGR5+ stem cells, but the transformed cells in the transit-amplifying compartment loose LGR5 expression while retaining high expression of β-catenin, as detected by IHC, and hence wnt signal activation, suggesting modulation of expression by other factors. In human cancer, overexpression of LGR5 has been noted in up to 90% of hepatocellular carcinomas with β-catenin mutations [61] and in a large proportion of colorectal carcinomas [34], [35], [60], where APC mutations and hence dysregulated wnt signalling are preponderant. Thus the elevated expression of LGR5 in colorectal cancer is likely to be secondary to dysregulated wnt signalling.

### LGR5 as a negative regulator of canonical wnt signalling and positive regulator of cell adhesion

Ablation of LGR5 in colorectal cancer cell lines results in a gene expression pattern consistent with increased canonical wnt signalling. Overexpression of LGR5 has the reverse effect. This observation, which is consistent with the ‘in vivo’ data presented by Garcia et al. showing that depletion of LGR5 causes persistent wnt signalling after it is normally switched off in the intestine [28], positions LGR5 as a negative regulator of canonical wnt pathways. Since LGR5 is a wnt-response gene, it is possible that we are observing a negative feedback loop where LGR5 expression keeps in check over-activation of canonical wnt signalling. Given the strong upregulation of wnt5a upon reduction in LGR5 levels, it is also likely that presence of LGR5 suppresses non-canonical wnt signalling. Interestingly, in our experiments many of the wnt response genes modulated by LGR5 expression are linked to EMT, e.g. collagens, fibronectin, MMPs, wnt5a and FGF4. These changes in expression pattern are associated with alterations in anchorage-independent proliferation, in invasion, migration, cell adhesion, tumourigenicity and tumour morphology with opposing phenotypes of LGR5 knockdown and LGR5 overexpression. Although our results show a switch of the cell lines to a more mesenchymal phenotype upon removal of LGR5, this transition does not have all the hallmarks of classical EMT: E-cadherin and vimentin expression levels are not markedly changed, however N-cadherin is elevated, and there is strong upregulation of EMT ‘mastermind’ genes ZEB, SNAIL and TWIST. Our data lead to the hypothesis that LGR5 expression in intestinal stem cells in the context of activated wnt signalling serves to restrict the stem cells to their niche, thus preventing inappropriate migration while maintaining selective aspects of wnt-driven anti-differentiation program. It is interesting, in this context, that most cell-surface markers of intestinal stem cells are adhesion molecules. This view accords with the statement of van den Brink and Offerhaus [62] in a recent review: “Appropriate inhibition of canonical WNT signalling not only acts to restrict the precursor-cell compartment to the precursor-cell niche within a single colonic crypt but also functions as an important brake on lateral stem-cell expansion through crypt fission”.

### Implications for colorectal carcinogenesis

The expression of LGR5 in intestinal stem cells, its dependence on activated wnt signalling, and its link to cell invasiveness position LGR5 as a potential player in the transition from adenoma to invasive carcinoma. In one study [60], similar positivity for β-catenin and LGR5 (28% vs. 27%) was observed in adenomas, but not in carcinomas (54% positive for LGR5 vs. 81% positive for β-catenin), suggesting that in carcinomas the expression of LGR5 may become disconnected from canonical wnt signalling, or that excess stimulation of wnt signalling may lead to loss of LGR5. Since LGR5 suppresses wnt signalling and reduces EMT, expression of LGR5 might be expected to be reduced during specific stages of colorectal carcinogenesis, particularly at the invasive front of the tumours. Two recent reports [63], [64] support a bi-modal regulation of LGR5 expression by wnt signalling: induction of LGR5 at medium levels of wnt activation but loss of expression with higher levels of wnt activation. Comparing two genetic mouse models of intestinal carcinogenesis, Lewis et al. [64] show highest LGR5 expression in tumours from APC(1322T) mice compared with APC(min) mice, with inverse correlation to β-catenin localization and the expression of wnt response genes, suggesting that LGR5 expression is stimulated within a relatively narrow range of wnt activation.[64]. In a different experimental system (endometrial cancer model) Sun et al [63] demonstrate that both overactivation of wnt signalling and absence of wnt signalling reduce the expression of LGR5 in the uterus. We have observed a similar phenomenon in colorectal carcinoma cell lines expressing heterozygous APC mutations, where exposure to increasing amounts of wnt3a leads to a dose-dependent increase of LGR5 RNA in the range of 20–70 ng/ml, but no significant increase in LGR5 is detected when wnt3a is used at 200 ng/ml. (Fig. S11) We propose that these findings have implications for the role of LGR5 in colorectal cancer progression: induction of wnt activity by APC mutation or β-catenin mutation would maintain LGR5 expression outside the stem cell niche, however overstimulation of wnt signalling at the invasive front of a tumour (where there is loss of E-cadherin and β-catenin is reported to be strongly nuclear: [65]) would be expected to reduce LGR5 expression, thus facilitating wnt-stimulated EMT and favouring invasiveness.

Thus the transition from adenoma to carcinoma may involve selective loss of LGR5 in areas of wnt hyperactivation, contributing to EMT and invasiveness. This hypothesis needs to be directly investigated by co-staining a large set of primary colorectal cancer specimens, including the invasive front, for LGR5, β-catenin, wnt pathway target proteins and markers of cell adhesion or EMT.

## Materials and Methods

### Reagents and antibodies

L-cells and L-cells expressing wnt 3a or wnt 5a were a kind gift from Dr Hong Zhou, ANZAC rResearch Institute, University of Sidney (ATCC # CRL-2648, CRL-2647 and CRL-2814 respectively).The ability of medium conditioned by cells expressing wnt3a to activate the canonical wnt signalling pathway was confirmed in β-catenin stabilization assays. Briefly, L-cells were exposed to control L-cell conditioned medium or wnt3a conditioned medium (each at 30% v/v) for 6 hr, then lysed in SDS sample buffer for SDS-PAGE of total proteins. The gels were transferred to nitrocellulose and probed with anti-β-catenin antibody. Increase in the level of β-catenin upon incubation with wnt3a reflects activation of canonical signalling.

Antibodies used in this study were: anti-LGR5 N-terminal region Sigma Prestige HPA012530 (Sigma, St.Louis, MO); anti-LGR5 central region, AP2745f (Abgent, SanDiego, CA); anti E-cadherin mouse monoclonal antibody #610182 (BD Transduction Laboratories, San Diego CA); anti β-catenin mouse monoclonal antibody #19920/610153 (BD Transduction Laboratories, San Diego CA); anti-CD44 rat monoclonal antibody #103002 (BioLegend, San Diego, CA) and AP00142PU-N (Novus Biologicals, Littleton, CO); anti flag antibody M2 (Sigma, St Louis, MO); anti ZO-1 antibody #61096 (BD Transduction Laboratories, San Diego CA). Secondary antibodies were anti-mouse Ig Alexa488,, anti-rat Ig Alexa488 and anti-rabbit Ig Alexa488, all from Invitrogen Molecular Probes (Eugene, OR); anti-mouseIg, and anti rabbit Ig IRDye800 and IRDye 700 were obtained from Li-Cor (Lincoln, Nebraska).

### Cell lines

LIM1899, Lim1215, LIM2537 and Lim1863 human colorectal carcinoma cell lines (CellBank, Sidney, Australia) were used in this study. Cells were routinely passaged in RPMI 1640 containing 10% foetal calf serum (FCS) with the following additives: hydrocortisone (1 µg/ml), thyoglycerol (0.01 µg/ml), and insulin (0.025 U/ml). LIM1899 stably expressing pTune/-LGR5 were maintained in RPMI1640+Adds+10% FCS with the addition of neomycin (G418) at a final concentration of 1.5 mg/ml. LIM1899 stably expressing non-target shRNA (NT) or shRNA to LGR5 (shLGR5) were maintained in RPMI1640+Adds+10% FCS with the addition of hygromycin at a final concentration of 2 µg/ml.

### mRNA and DNA preparation

mRNA was isolated from cells with an Illustra RNAspin Mini RNA isolation Kit (GE Healthcare, Cat. No. 25-0500-71). To preserve RNA stability, samples were kept frozen in a −80°C freezer. cDNA was prepared by reverse transcription from RNA using High-Capacity cDNA Reverse Transcription Kits (Applied Biosystem, cat#. 4368814).

### Wnt stimulation

Cells were plated in 6-well trays (for RNA preparation) or in Lab-Tek microchamber slides (Nunc, Rochester, NY) and grown to approximately 80% confluence. Culture medium, L-cell conditioned medium or medium conditioned by L-cells expressing wnt3a or wnt5a were added to parallel wells to a final concentration of 30% (v/v). Cells were harvested at different time points for RNA preparation or for immunofluorescence analysis.

In wnt3a titration experiments recombinant wnt3a (R&D Systems, MN,USA) was used instead of wnt3a conditioned medium.

### Immunofluorescence

Cultured cells were plated onto LabTek microchamber slides a minimum of three days before processing. Frozen sections (5 µm) obtained from mouse xenografts of LIM1899 cells were air dried on glass slides for 1 hr before processing.

All slides were fixed in 4% formaldehyde/PBS for 15 min, and permeabilized in 0.2%Triton X-100 in PBSfor 10 min. Non specific binding sites wee blocked by incubation with 2% Bovine Serum Albumin (BSA) in PBS for 1–3 hr at RT. Slides were incubated with the relevant primary antibody (1 hr at RT or overnight at 4°C), washed three times and incubated with Alexa 488 or Alexa 546-labelled secondary antibodies (45 min at RT). In some experiments, rhodamine-labelled phalloidin was included with the secondary antibody. DAPI (0.1 µg/ml; Molecular Probes) was added in the last ten minutes of incubation. Slides were dehydrated sequentially in Ethanol and Xylene and mounted with DPX mounting medium. Samples were imaged with Nikon Plan Apo 60× (NA1.4) oil immersion lens on a Nikon C1 confocal microscope, .or in wide field with a Nikon 90i fluorescent microscope. Images were obtained using standard filter sets and laser lines.

### Immunoblotting

Cells in culture wells were rinsed 1× in ice-cold PBS then scraped directly in Cell Lysis Buffer (CLB:HEPES pH7.5 20 mM, NaCl 150 mM, EDTA 5 mM, Triton X-100 1%v/v, Sodium Deoxycholate 1% v/v, protease inhibitors) on ice. The lysates were incubated for 45 min at 4°C, then centrifuged at 13,000 rpm for 30 min at 4°C. Supernatant fluids and pellets were harvested separately. Both samples were resuspended in SDS sample buffer and boiled for 10 min. Proteins were separated on 4–12% Bis-Tris gradient gels (Invitrogen, Carlsbad CA) and transferred electrophoretically onto nitrocellulose membranes using a iBlot dry transfer system (Invitrogen, Carlsbad, CA). The membranes were incubated in Tris-buffered saline (TBS) containing skim milk powder (3%w/v) for 1–3 hrs at RT to block non-sppecific binding sites, then incubated with the appropriate antibodies for 3 hrs at RT or 16 hrs at 4°C. Fluorescent secondary antibodies were purchased from Li-Cor (Lincoln, NB). Immunoreactive bands were detected using Odyssey infrared photometer (Li-Cor, Lincoln, Nebraska) according to the manufacturer's protocol. Quantitation was performed in ImageQuant using wide-band integration.

### FACS analysis

Cells were harvested by trypsinization, collected in FACS buffer (PBS containing 5%v/v FCS and 5 mM EDTA), and aliquoted in individual tubes for antibody staining. Cells were incubated with the appropriate first antibody for 40 min on ice, washed twice in ice-cold PBS, then incubated with the appropriate Alexa 488-coupled secondary antibody. Fluorescence profiles were acquired on a BD FACSCalibur (Franklin Lakes, NJ) using the CellQuest program.

### Silencing of LGR5

siRNAs (Ambion, Austin, TX) and shRNAs (Sigma, St Louis, MO) were used to silence LGR5 and Msi-1 expression. For shRNA delivery, the cells were infected with lentiviral particles encoding shRNA to Lgr5, Msi-1 or non-target as per manufacturer instruction. Stable cell lines were established with puromycin selection. Cy3 labeled Lgr5-siRNA (Ambion, Cat No AM16811, ID 139290, Austin, TX, USA) and vector control (Ambion, Cat No. AM4624) were used for transient transfections Cells for transfection were plated one day prior to transfection at 4×10^5^ cell/well in 6 well plates. For preparation of the transfection reagent 15 µL X-tremeGENE siRNA transfection reagent were mixed with 285 µL of serum-free Opti-MEM-1 medium, and in a second tube 48 µL CY3 labelled LGR5-siRNA or vector control were mixed with 252 µL OPT medium. The contents of the two tubes were mixed and incubated for 15 minutes at room temperature. 600 µL of mixture was added for each well in 2.5 mL medium without antibiotics and 10% FCS. The cells were incubated at 37°C in an atmosphere of 10%CO_2_ for the appropriate time (1–5 days) before harvesting.

### Overexpression of LGR5

Cells were plated one day prior to the transfection experiment at 4×10^5^ cell/well in 6 well plates. Cells were transfected with the pTUNE Inducible Vector without insert (vector control) or containing the LGR5 ORF construct flanked by Flag (DDK) and Myc sequences (OriGene, Cat. No. RT212825) using FuGENE HD transfection Reagent (Roche, Cat. No. 04709713001). The LGR5 construct was fully sequenced prior to use, and the sequence proven to be correct. Transfection complex was prepared by adding 6 µg plasmid DNA in 92 µL OPT medium followed by addition of 8 µL FuGENE HD Reagent (ratio of 8∶6). The mixture was incubated at room temperature for 15′, then 100 µL of mix were added to each well. After 72 h–120 h culture at 37°C, the cells were harvested for protein and RNA isolation. Stable cell lines were derived by selection in Neomycin (G418, 1.6 mg/ml). Mock-transfected LIM1899 cells, exposed to the transfection reagent but in the absence of DNA, were used as a control.

### qRT-PCR and Superarrays

RNA was prepared form each cell line with RNeasy Plus mini kit (Qiagen). cDNA was synthesized from 5–20 µg RNA per sample using a high capacity cDNA reverse transcription kit (Applied Biosystems). qRT-PCR was performed using the primers listed in Table S7. PCRs were carried out in a reaction volume of 25 µl using Power SYBR Green PCR Master Mix (AppliedBiosystems). GAPDH was used as internal control. The samples were amplified in a in 7300 Real-Time PCR system (Applied Biosystem) and data analysed with SDS software version 4.0 (Applied Biosystem) using the ΔΔCT method.

RT2 Profiler™ PCR arrays (Superarrays) and qPCR MasterMix were purchased from SABioscience (Qiagen, Germantown, MD, USA). Each array was performed in triplicate according to the manufacturer's instructions using 5 µg of cDNA per plate. Results were analysed with ABI 7300 SDS software (Applied Biosystems) and SaBioscience RT2 data analysis program. All dissociation curves were checked manually, and samples with double peaks eliminated from the analysis. Baseline and threshold values were set manually for each detector.

### Cellular assays

#### Proliferation (MTT)

Cell proliferation was measured using an MTT assay. Cells were counted, resuspended at 2×10^3^ cells per 200 µl medium, and plated in 96-well plates for incubation at 37°C and 10%CO_2_ in air for up to 10 days. At the end of the incubation, 10 µl of 5 um MTT reagent (Sigma, St Louis, MO, USA) was added to each well, and incubated for 4 h. The plates were centrifuged at 1500 rpm for 10 min and the medium were removed. The MTT precipitate was solubilised by the addition of 200 µl/well acidified isopropanol, and the absorbance at 570 nm was measured in triplicate wells.

#### Soft agar cloning

Cells were counted, resuspended at 2.5×10^3^/ml or 5×10^2^/ml in medium (DME with 10% FBS and L-glutamine) containing 0.3% w/v agar (Bacto, Duckinson, Sparks MD, USA) and overlayed onto a 30-mm dish containing a solidified bottom layer of 0.6% w/v agar in the same medium. After incubation for 10–15 days at 37°C and 10%CO_2_, all dishes were stained by adding 1 ml/dish of 0.01%(w/v) crystal violet (Fronine, Taren Point, NSW, Australia) and the colonies counted with a dissecting microscope. Assays were performed in triplicate.

#### Spheroid cultures

Cells were trypsinized, washed in complete medium and resuspended in RPMI+Adds with 20% FCS at 1.66×10^4^ cells/ml. Culture dishes were filled with PBS; 30 µl droplets (500 cells/drop) were deposited on the up-turned inner surface of the lid, then the lid was then inverted and placed on top of the culture dish. The hanging drops were cultured for up to 10 days in 37°C incubator with 10%CO_2_. The resulting spheroids were photographed in phase contrast on a Nikon Eclipse Ti microscope using a 10× lens and a with a Colormetrics Coolsnap HQ ccd camera. The volume of the sphe*roids was estimated using* the modified ellipsoid formula (1/2 length×width^2^) using 10 individual images per cell line. The average cell number of the spheroid was measured in 10 spheroids per cell line: the spheroids were individually trypsinized to yield single cell suspensions, and the cell number counted using a hemocytometer.

#### Wound repair assays

Cells were plated in 24-well plates at 10^6^ cells/well in 1 ml culture medium. Two days later a wound was scratched in the adherent cell monolayers with an Eppendorf tip and the medium was changed to remove loosened cells. The wells were examined every two days and photomicrographs taken on a Nikon Eclipse Ti as described above. Wound width was measured on photomicrographs, using the same area of the well for each measurement.

#### Transwell assays

Cell culture inserts (8 µ pore size, cat#353097, BD Falcon, Franklin Lakes NJ) were placed in 24-well plates containing 1 ml of culture medium and the chambers seeded with 5×10^5^ cells in 300 µl culture medium. Plates were incubated for three days before changing the medium in the upper chamber to serum-free RPMI. Cells were incubated for a further 24 h before harvesting the filters. To stain the filters, all medium was removed from both the wells and the inserts and replaced with 1 ml of May-Grumwald reagent for 4 min. After rinsing extensively in tap water, the filters were counterstained in Giemsa (1∶40) for 7 min and again washed in tap water. Cells attached to the upper side of the filters were scraped using a cotton swab, rinsed twice and re-swabbed. Filters were excised from the inserts and mounted on glass slides with the lower side (containing the migrating cells) towards the glass. Cells were counted using a light microscope with a 10× lens.

### Tumour Xenografts

LIM1899 cells were grown in bulk and transfected with either pTune-LGR5 vector, control vector or Cy3-siRNA to LGR5 as described above. Two days after transfection the cells were collected by trypsinization, counted and injected at 5×10^6^ cells/site subcutaneously on both flanks of nude mice. Tumours were measured twice weekly with callipers. Tumour volumes were estimated using the modified ellipsoid formula V = 1/2 (length×width^2^). Tumour mass was determined by weighing each dissected tumour at the end of the experiment.

## Supporting Information

Figure S1 **Specificity of LGR5 staining and induction by wnt 3a.** **A**): HEK293T cells were transiently transfected with a construct encoding for flag-tagged LGR5 as described in Methods. Cells were processed for immunofluorescence and stained with rhodamine-phallodin (red channel), DAPI (blue channel) and commercially available antibodies to the flag tag (M2) or to LGR5 (Ap2745f and HPA012530) followed by Alexa 488 secondary antibody (green channel), as described in Methods. Left panels: untransfected cells; right panels: cells transfected with M2-LGR5. **B**): LIM2537 and LIM1863 cells were incubated for 48 hrs with control medium (L-cell conditioned medium) or with conditioned medium from wnt3a-transfected L-cells. Cells were prepared for immunofluorescence and stained with rhodamin-phalloidin (red channel), DAPI (blue channel) and anti-LGR5 antibody Ap2745f followed by Alexa488 anti-rabbit Ig (green channel).Left panels: composite image (three channels); right panels: LGR5 staining (green channel) only. In the same experiment, exposure to wnt 5a did not alter the levels of LGR5 detectedable by IF. Cells were imaged on a Nikon C1 confocal microscope using a 60× oil lens. Laser gains were set on negative control slides (irrelevant primary antibody) and kept constant throughout. Images were processed using EZ-C1 software.
(TIF)

Figure S2 **Confocal analysis of LGR5 staining after silencing of LGR5.** LIM1215 and Lim1899 cells expressing non-target shRNA or shRNA to LGR5 were grown in microchamber slides and prepared for immunofluorescence as described in Methods. Cells were stained with anti E-cadherin antibody followed by Alexa 546 anti-mouse Ig (red), anti-LGR5 followed by Alexa 488 anti-rabbit Ig (green) and the nuclear stain DAPI (blue). Left panels: composite image (three channels); right panels: LGR5 staining only (channel 2, greyscale). Images were acquired and processed as described in the legend to Fig. S1.
(TIF)

Figure S3 **Cell proliferation in adherent cell cultures.** Cells expressing various constructs were tested for their ability to proliferate under standard tissue culture conditions using the MTT assay as described in Methods. **A**: LIM 1215 cells (left panel) and LIM1899 cells (right panel) were either not transfected (parental), or transduced with lentiviral shRNA to non-target sequences (NT),to LGR5 (shLGR5) or to Msi-1 (shMsi-1). Cells containing the shRNAs were selected for one week in puromycin, then switched to normal medium for three days before assay. Specific knockdown of LGR5 and Msi-1 was confirmed by qRT-PCR in parallel samples. **B**: LIM1899 cells were mock-transfected (parental), transfected with empty pTune vector (vector), or transfected with pTune vector containing LGR5 (LGR5). Cells were grown for three days after transfection then assayed. Overexpression of LGR5 was confirmed by qRT-PCR on parallel samples.
(TIF)

Figure S4 **Stable LGR5 overexpression in LIM1899-derived cell lines.** LIM1899 cells were transfected with pTune/LGR5 and selected for expression of the construct in medium containing neomycin. Stable cell lines were expanded, switched to antibiotic-free medium and characterized for LGR5 expression and clonogenicity. **A**) LGR5 expression by immunofluorescence: parental cells and three clonal cell lines overexpressing LGR5 cells were fixed, permeabilized and stained with anti-flag antibody (M2) followed by Alexa 488 anti-mouse Ig (green) and nuclear stain DAPI (blue). Images were collected and analysed as described in Methods. **B**) Expression of LGR5 in parental cells and stable cell lines was determined by qRT-PCR. Parental LIM1899 mRNA was used as an equalizer. Data are the average and sd of duplicate determinations. **C**) Clonogenicity in soft agar: cells were seeded in soft agar plates and colony numbers determined after 10 days as described in Methods. Results are presented as mean values of each test sample over control (untransfected) cells. Each cell line was tested in triplicate. Statistical significance was determined by unpaired t-test. ** = p<0.005 *** = p<0.001. **D**) Correlation between expression of LGR5 and loss of clonogenicity in soft agar. The data presented in graphs B and C were plotted against each other, and fitted using a first-order exponential decay function.
(TIF)

Figure S5 **Quantitation of cellular proteins in LIM1899 with altered expression of LGR5.** **A**) Total cellular lysates from untransfected LIM 1899 (parental) and LIM1899 transfected with vector (V), with siLGR5 or with pTune/LGR5(LGR5) were analysed by SDS-PAGE and immunoblotting as described in Methods. In some experiments, both transient (LGR5 T) and stable (LGR5 S) transfectants of pTune/LGR5 were tested in parallel. There was no appreciable difference in protein expression between transient and stable LGR5 transfectants, and the results have been pooled in the quantitative analysis. **B**) Quantitation of protein expression from immunoblotting experiements. Band intensity was quantitated by wide-line integration using ImageQuant. The relative amount of each protein is expressed as a ratio of the specific band to the loading control β-tubulin for each lane. The data are presented as average and sd of at least three transfection experiments analysed on separate gels. To make the data from each gel comparable, all ratios have been normalized setting the value of the protein level in the parental cell line in each experiment to 1.
(TIF)

Figure S6 **Expression of CD antigens on parental and transfected LIM1899 cells.** LIM1899 cells were transfected with Cy3 siRNA to LGR5 (siLGR5) or with pTune/LGR5 (LGR5 Tr). Three days after transfection parental cells and transfected cells were harvested and processed for FACS analysis as detailed in Methods. **A**): Histograms of fluorescence profiles of cells stained with CD44, CD133 and CD166 antibodies. Solid purple = parental cells; red overlay = LGR5 Tr cells; teal overlay = siLGR5 cells; green overlay = negative antibody control. **B**): Median fluorescence channel values for each sample. Data were acquired on a FACS Calibur instrument and analysed using the CellQuest program.
(TIF)

Figure S7 **CD44 distribution in LIM 1215 cells after silencing of LGR5.** LIM1215 cells were transduced with either non-target shRNA (NT) or shRNA to LGR5 (shLGR5). Cells were seeded in chamber slides, fixed and stained with rhodamine-phalloidin (red channel), anti-CD44 followed by Alexa 488 anti-rat Ig (green channel) and nuclear stain DAPI (blue channel). Top panels: composite image with three channels. Bottom panels: grey scale image for CD44.
(TIF)

Figure S8 **Wnt array.** mRNA was prepared from LIM1899 untransfected, transfected with empty vector, with siLGR5 or transfected with pTune/LGR5. Expression of genes in the wnt pathway was determined by qRT-PCR using Superarray plates (SABioscience). The experiments were performed and analyzed as described in Methods. Data are the mean of three independent experiments for each data set. “Parental” set includes untransfected cells and cells transfected with empty vectors. Left panels show the correlation in gene expression levels between parental (abscissa) and test (ordinate) samples. Right panels show the “volcano plots” of expression changes (abscissa) vs statistical significance (ordinate) The black line indicates no change (fold change = 1), the red lines indicate the 2-fold change threshold, and the blue line in the volcano plots indicates the p value chosen for t-test threshold. **A**) LGR5 overexpressors vs control cells; **B**) LGR5 knockdown vs control cells.
(TIF)

Figure S9 **Notch array.** mRNA was prepared from LIM1899 untransfected, transfected with empty vectors, with siLGR5 or with pTune/LGR5. Expression of genes in the Notch pathway was determined by qRT-PCR using Superarray plates (SABioscience). The experiments were performed and analyzed as described in Methods. Data are the mean of three independent experiments for each data set. “parental” set includes untransfected cells and cells transfected with empty vectors. Left panels show the correlation in gene expression levels between parental (abscissa) and test (ordinate) samples. Right panels show the “volcano plots” of expression changes (abscissa) vs statistical significance (ordinate) The black line indicates no change (fold change = 1), the red lines indicate the 2-fold change threshold, and the blue line in the volcano plots indicates the p value chosen for t-test threshold. **A**) LGR5 overexpressors vs control cells; **B**) LGR5 knockdown vs control cells.
(TIF)

Figure S10 **EMT array.** mRNA was prepared from LIM1899 untransfected, transfected with empty vectors, with siLGR5 or with pTune/LGR5. Expression of genes in the EMT pathway was determined by qRT-PCR using Superarray plates (SABioscience). The experiments were performed and analyzed as described in Methods. Data are the mean of three independent experiments for each data set. “parental” set includes untransfected cells and cells transfected with empty vectors. Left panels show the correlation in gene expression levels between parental (abscissa) and test (ordinate) samples. Right panels show the “volcano plots” of expression changes (abscissa) vs statistical significance (ordinate) The black line indicates no change (fold change = 1), the red lines indicate the 2-fold change threshold, and the blue line in the volcano plots indicates the p value chosen for t-test threshold. **A**) LGR5 overexpressors vs control cells; **B**) LGR5 knockdown vs control cells.
(TIF)

Figure S11 Colorectal cancer cell lines LIM2405 and LIM 2550 plated in 6-well trays were exposed to increasing concentrations of recombinant wnt 3a (0, 22, 66 or 200 ng/ml) and harvested 12 or 24 hrs after addition of the stimulus. mRNA was prepared from each well and the amount of LGR5 message quantitated by qRT-PCR. Duplicate wells were used for each condition, and the experiment was repeated twice. The graphs show the average and standard deviation of duplicate experiments as fold-change in LGR5 expression relative to the internal control (no wnt3a at 12 and 24 hr, respectively).
(TIF)

Table S1 Wnt Array. Changes in LIM1899 gene expression with upregulation of LGR5.
(DOC)

Table S2 Wnt array. Changes in LIM1899 gene expression with knockdown of LGR5.
(DOC)

Table S3 Notch Array. Changes in LIM1899 gene expression with overexpression of LGR5.
(DOC)

Table S4 Notch Array. Changes in LIM1899 gene expression with knockdown of LGR5.
(DOC)

Table S5 EMT Array. Changes in LIM1899 gene expression with overexpression of LGR5.
(DOC)

Table S6 EMT Array. Changes in LIM1899 gene expression with knockdown of LGR5.
(DOC)

Table S7 Primer list for quantitative real-time PCR.
(DOC)
