# Supplementary figures and images for: LGR5 Is a Negative Regulator of Tumourigenicity, Antagonizes Wnt Signalling and Regulates Cell Adhesion in Colorectal Cancer Cell Lines
Source: PLoS One. 2011 Jul 28;6(7):e22733. doi: 10.1371/journal.pone.0022733 (PMC3145754; doi:10.1371/journal.pone.0022733)

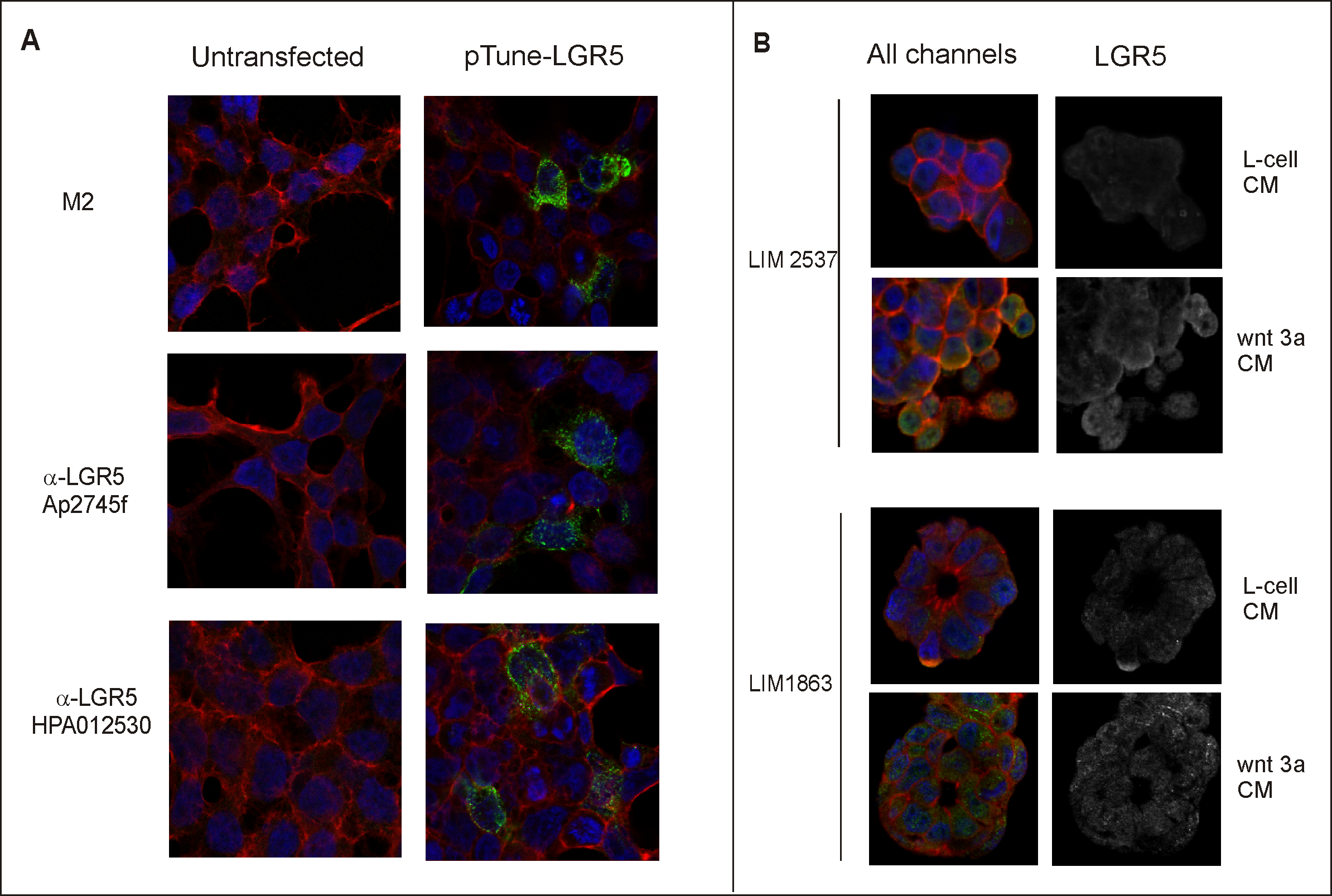

Supplement: Figure S1 — Specificity of LGR5 staining and induction by wnt 3a. A): HEK293T cells were transiently transfected with a construct encoding for flag-tagged LGR5 as described in Methods. Cells were processed for immunofluorescence and stained with rhodamine-phallodin (red channel), DAPI (blue channel) and commercially available antibodies to the flag tag (M2) or to LGR5 (Ap2745f and HPA012530) followed by Alexa 488 secondary antibody (green channel), as described in Methods. Left panels: untransfected cells; right panels: cells transfected with M2-LGR5. B): LIM2537 and LIM1863 cells were incubated for 48 hrs with control medium (L-cell conditioned medium) or with conditioned medium from wnt3a-transfected L-cells. Cells were prepared for immunofluorescence and stained with rhodamin-phalloidin (red channel), DAPI (blue channel) and anti-LGR5 antibody Ap2745f followed by Alexa488 anti-rabbit Ig (green channel).Left panels: composite image (three channels); right panels: LGR5 staining (green channel) only. In the same experiment, exposure to wnt 5a did not alter the levels of LGR5 detectedable by IF. Cells were imaged on a Nikon C1 confocal microscope using a 60× oil lens. Laser gains were set on negative control slides (irrelevant primary antibody) and kept constant throughout. Images were processed using EZ-C1 software. (TIF) [file pone.0022733.s001.tif]

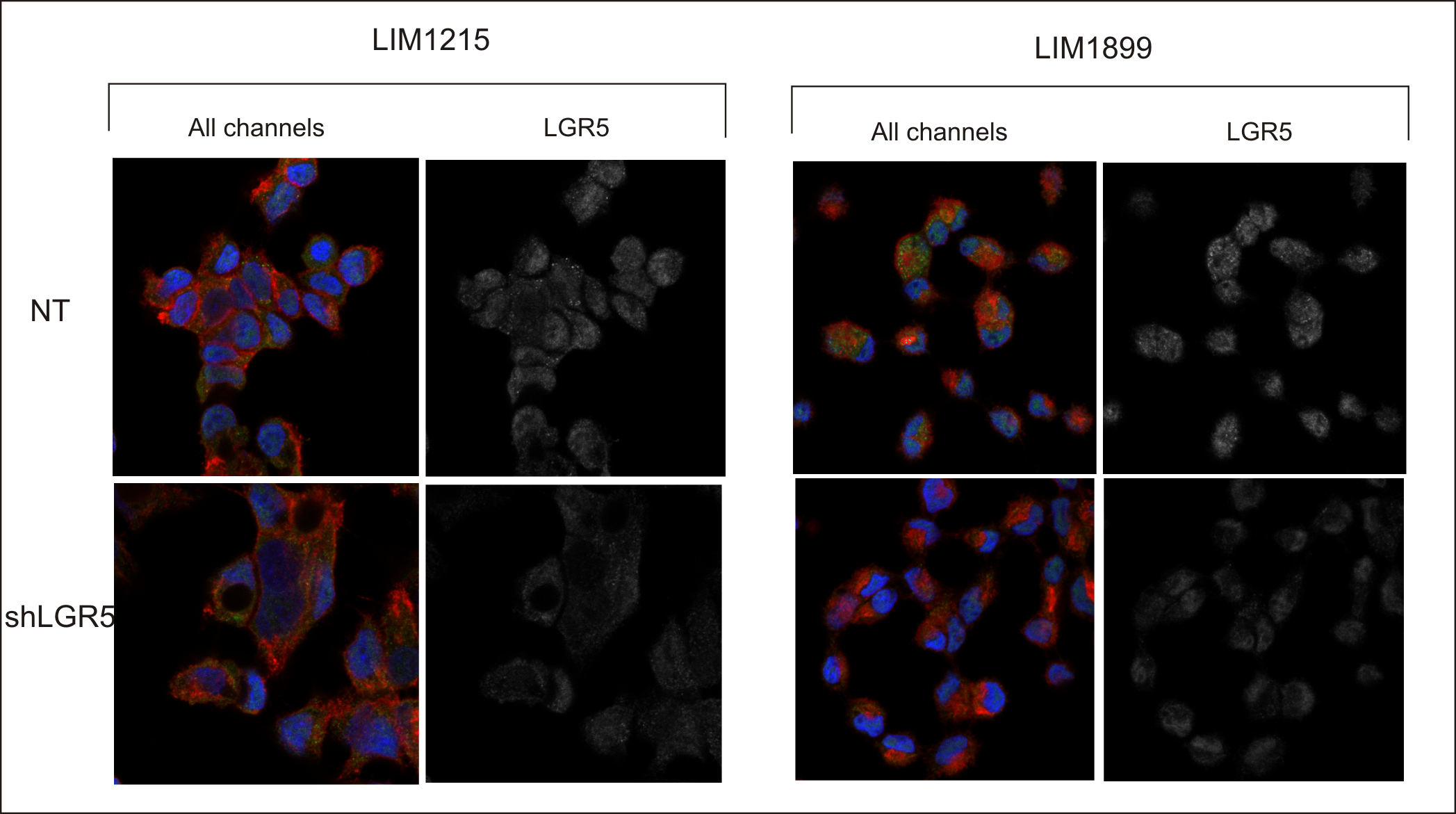

Supplement: Figure S2 — Confocal analysis of LGR5 staining after silencing of LGR5. LIM1215 and Lim1899 cells expressing non-target shRNA or shRNA to LGR5 were grown in microchamber slides and prepared for immunofluorescence as described in Methods. Cells were stained with anti E-cadherin antibody followed by Alexa 546 anti-mouse Ig (red), anti-LGR5 followed by Alexa 488 anti-rabbit Ig (green) and the nuclear stain DAPI (blue). Left panels: composite image (three channels); right panels: LGR5 staining only (channel 2, greyscale). Images were acquired and processed as described in the legend to Fig. S1. (TIF) [file pone.0022733.s002.tif]

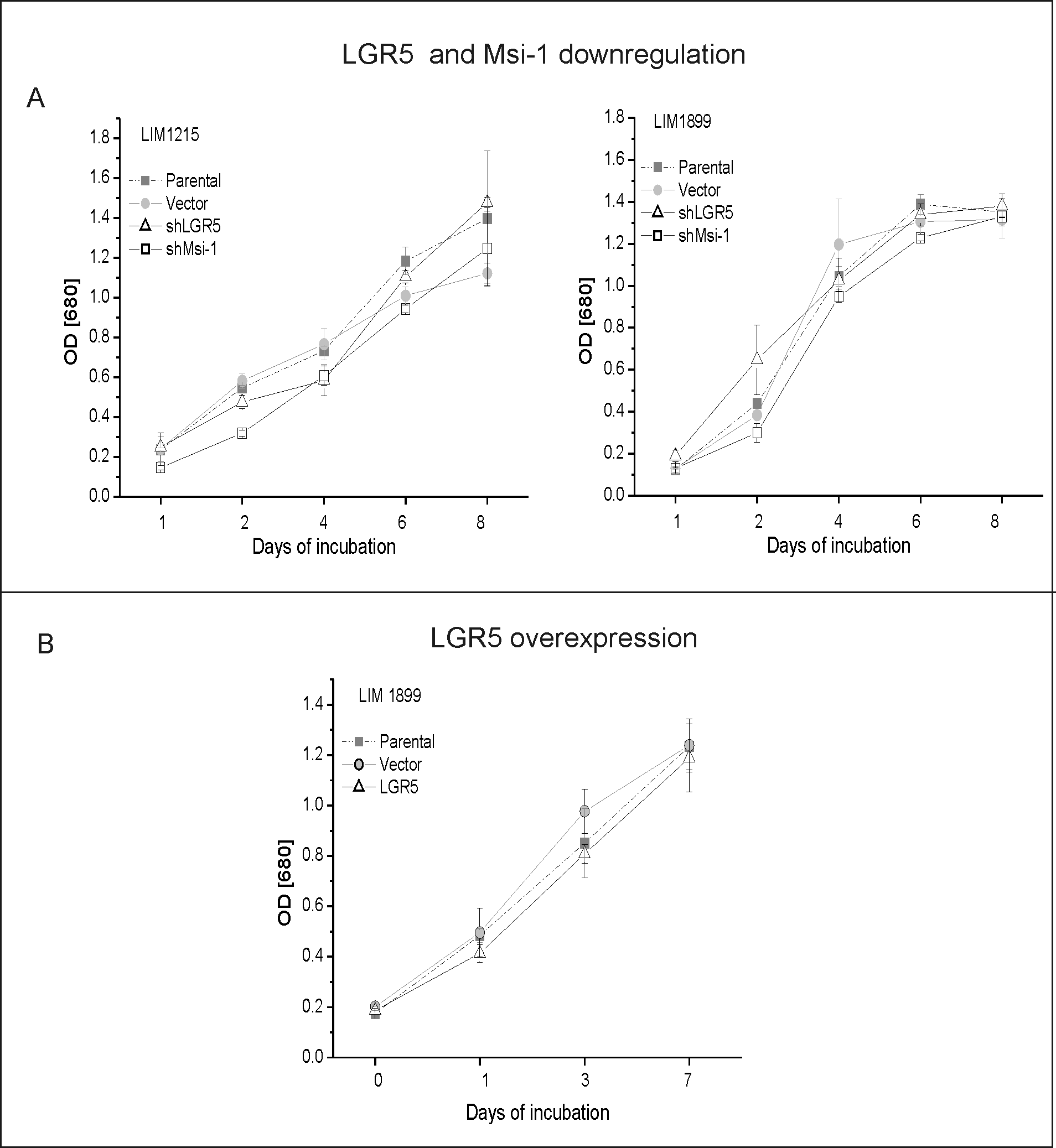

Supplement: Figure S3 — Cell proliferation in adherent cell cultures. Cells expressing various constructs were tested for their ability to proliferate under standard tissue culture conditions using the MTT assay as described in Methods. A: LIM 1215 cells (left panel) and LIM1899 cells (right panel) were either not transfected (parental), or transduced with lentiviral shRNA to non-target sequences (NT),to LGR5 (shLGR5) or to Msi-1 (shMsi-1). Cells containing the shRNAs were selected for one week in puromycin, then switched to normal medium for three days before assay. Specific knockdown of LGR5 and Msi-1 was confirmed by qRT-PCR in parallel samples. B: LIM1899 cells were mock-transfected (parental), transfected with empty pTune vector (vector), or transfected with pTune vector containing LGR5 (LGR5). Cells were grown for three days after transfection then assayed. Overexpression of LGR5 was confirmed by qRT-PCR on parallel samples. (TIF) [file pone.0022733.s003.tif]

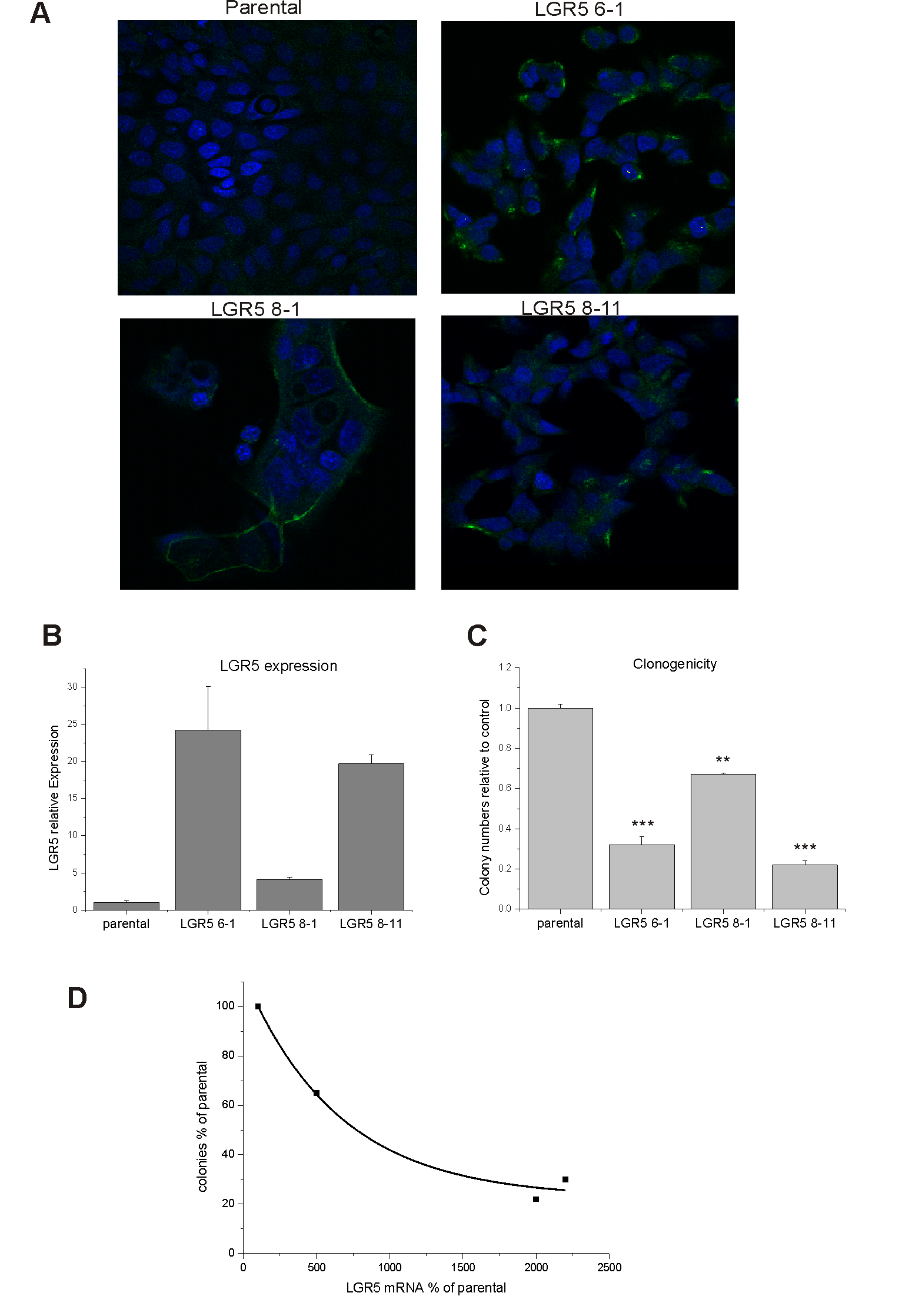

Supplement: Figure S4 — Stable LGR5 overexpression in LIM1899-derived cell lines. LIM1899 cells were transfected with pTune/LGR5 and selected for expression of the construct in medium containing neomycin. Stable cell lines were expanded, switched to antibiotic-free medium and characterized for LGR5 expression and clonogenicity. A) LGR5 expression by immunofluorescence: parental cells and three clonal cell lines overexpressing LGR5 cells were fixed, permeabilized and stained with anti-flag antibody (M2) followed by Alexa 488 anti-mouse Ig (green) and nuclear stain DAPI (blue). Images were collected and analysed as described in Methods. B) Expression of LGR5 in parental cells and stable cell lines was determined by qRT-PCR. Parental LIM1899 mRNA was used as an equalizer. Data are the average and sd of duplicate determinations. C) Clonogenicity in soft agar: cells were seeded in soft agar plates and colony numbers determined after 10 days as described in Methods. Results are presented as mean values of each test sample over control (untransfected) cells. Each cell line was tested in triplicate. Statistical significance was determined by unpaired t-test. ** = p<0.005 *** = p<0.001. D) Correlation between expression of LGR5 and loss of clonogenicity in soft agar. The data presented in graphs B and C were plotted against each other, and fitted using a first-order exponential decay function. (TIF) [file pone.0022733.s004.tif]

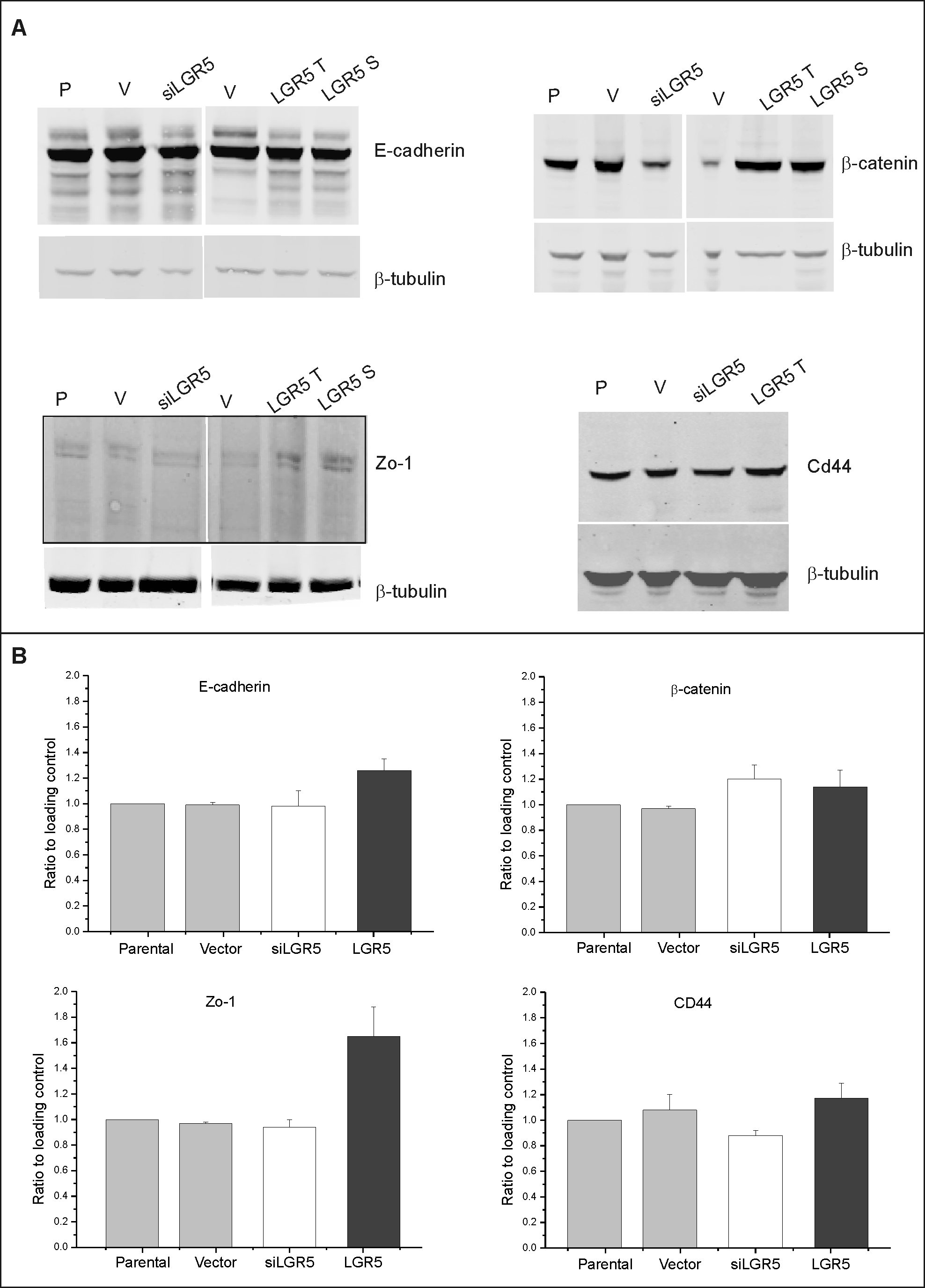

Supplement: Figure S5 — Quantitation of cellular proteins in LIM1899 with altered expression of LGR5. A) Total cellular lysates from untransfected LIM 1899 (parental) and LIM1899 transfected with vector (V), with siLGR5 or with pTune/LGR5(LGR5) were analysed by SDS-PAGE and immunoblotting as described in Methods. In some experiments, both transient (LGR5 T) and stable (LGR5 S) transfectants of pTune/LGR5 were tested in parallel. There was no appreciable difference in protein expression between transient and stable LGR5 transfectants, and the results have been pooled in the quantitative analysis. B) Quantitation of protein expression from immunoblotting experiements. Band intensity was quantitated by wide-line integration using ImageQuant. The relative amount of each protein is expressed as a ratio of the specific band to the loading control β-tubulin for each lane. The data are presented as average and sd of at least three transfection experiments analysed on separate gels. To make the data from each gel comparable, all ratios have been normalized setting the value of the protein level in the parental cell line in each experiment to 1. (TIF) [file pone.0022733.s005.tif]

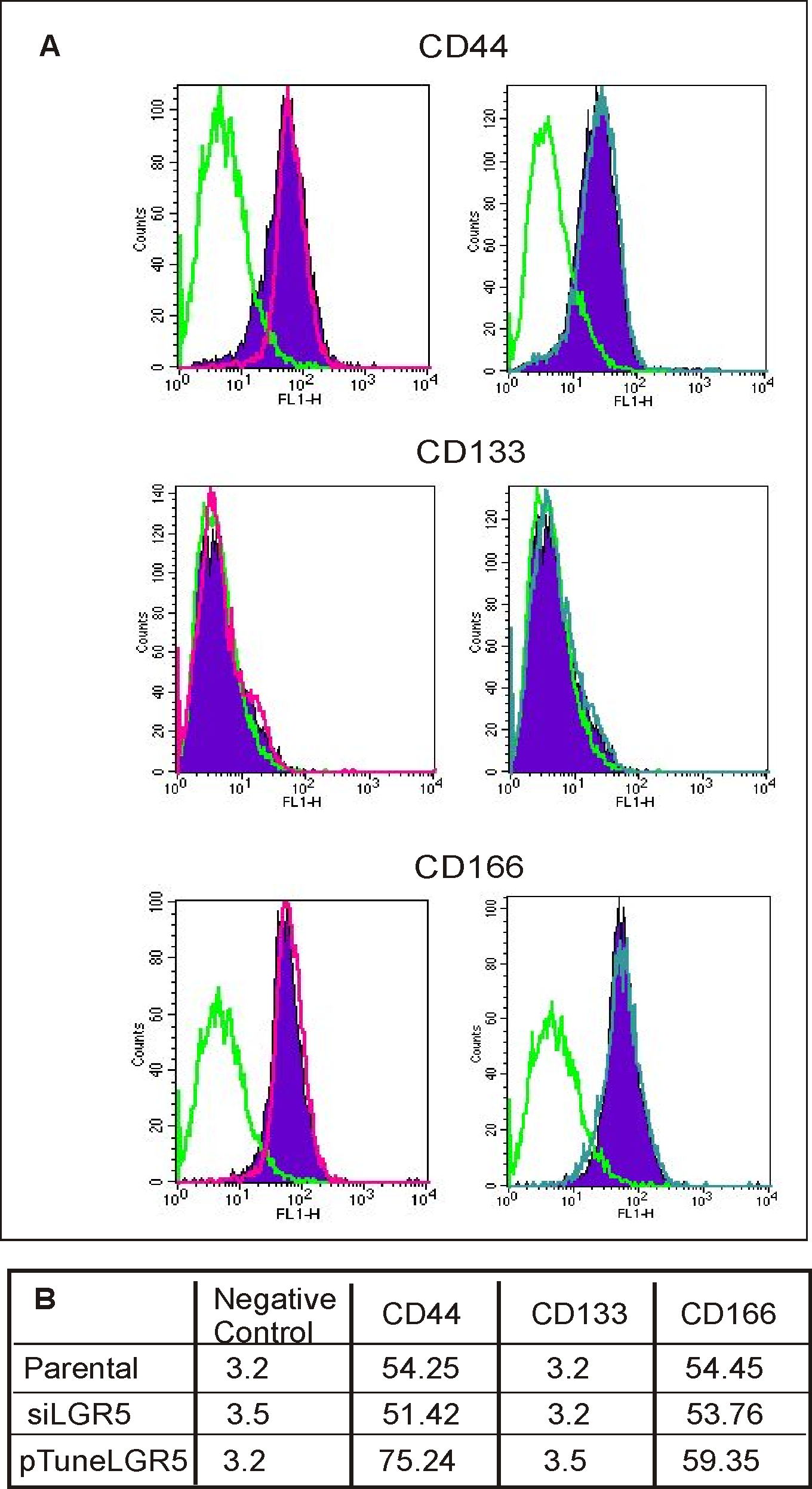

Supplement: Figure S6 — Expression of CD antigens on parental and transfected LIM1899 cells. LIM1899 cells were transfected with Cy3 siRNA to LGR5 (siLGR5) or with pTune/LGR5 (LGR5 Tr). Three days after transfection parental cells and transfected cells were harvested and processed for FACS analysis as detailed in Methods. A): Histograms of fluorescence profiles of cells stained with CD44, CD133 and CD166 antibodies. Solid purple = parental cells; red overlay = LGR5 Tr cells; teal overlay = siLGR5 cells; green overlay = negative antibody control. B): Median fluorescence channel values for each sample. Data were acquired on a FACS Calibur instrument and analysed using the CellQuest program. (TIF) [file pone.0022733.s006.tif]

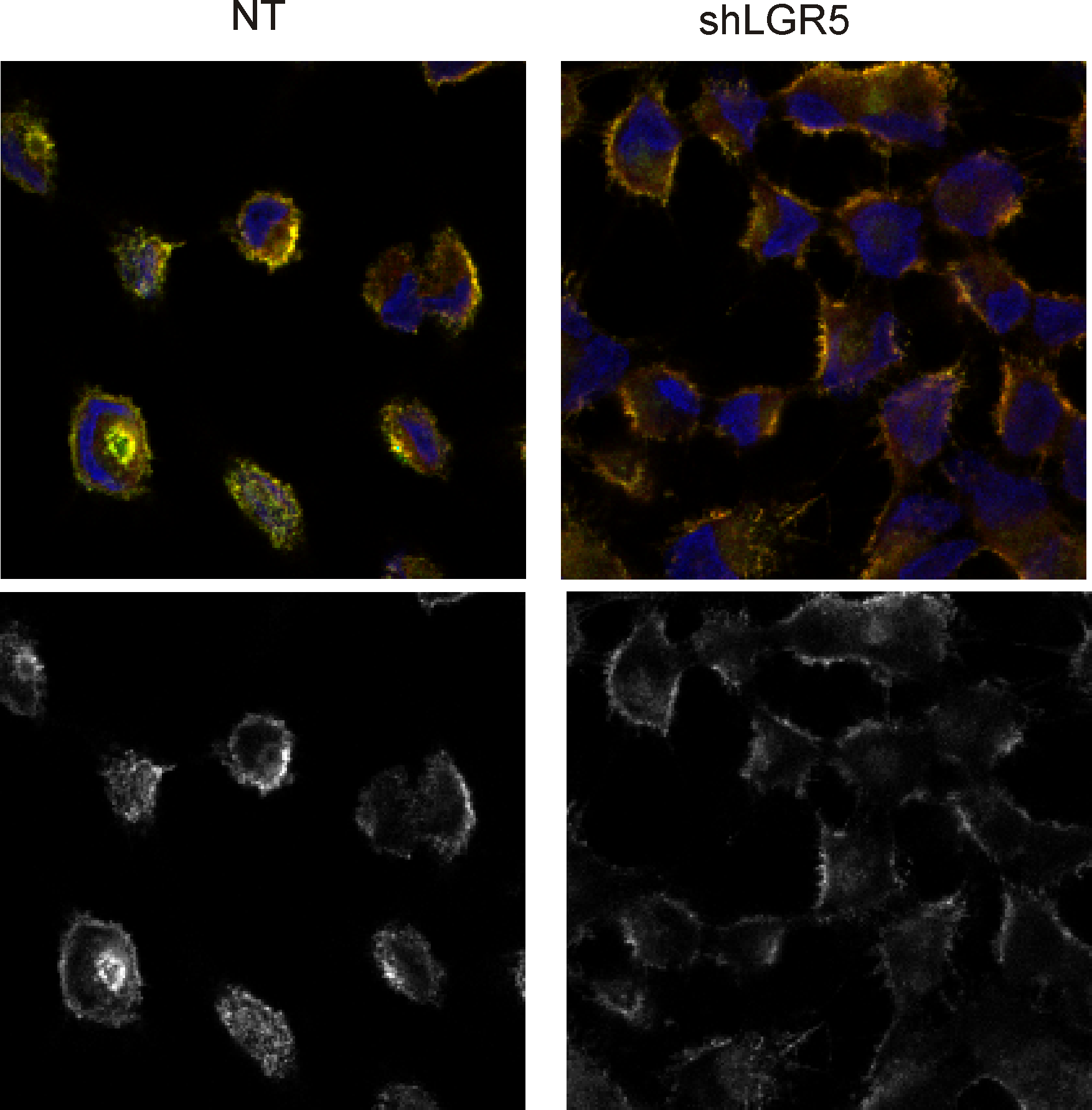

Supplement: Figure S7 — CD44 distribution in LIM 1215 cells after silencing of LGR5. LIM1215 cells were transduced with either non-target shRNA (NT) or shRNA to LGR5 (shLGR5). Cells were seeded in chamber slides, fixed and stained with rhodamine-phalloidin (red channel), anti-CD44 followed by Alexa 488 anti-rat Ig (green channel) and nuclear stain DAPI (blue channel). Top panels: composite image with three channels. Bottom panels: grey scale image for CD44. (TIF) [file pone.0022733.s007.tif]

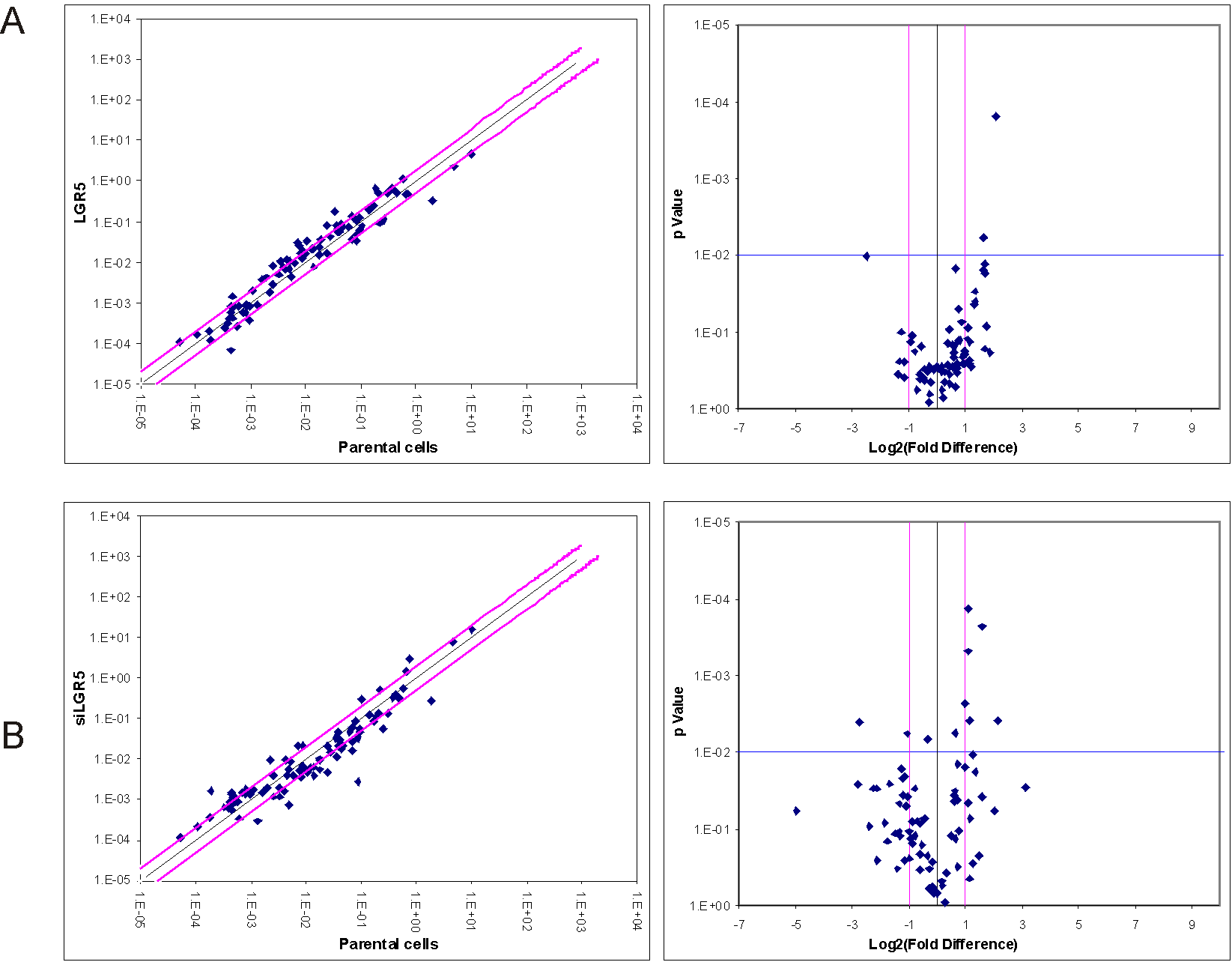

Supplement: Figure S8 — Wnt array. mRNA was prepared from LIM1899 untransfected, transfected with empty vector, with siLGR5 or transfected with pTune/LGR5. Expression of genes in the wnt pathway was determined by qRT-PCR using Superarray plates (SABioscience). The experiments were performed and analyzed as described in Methods. Data are the mean of three independent experiments for each data set. “Parental” set includes untransfected cells and cells transfected with empty vectors. Left panels show the correlation in gene expression levels between parental (abscissa) and test (ordinate) samples. Right panels show the “volcano plots” of expression changes (abscissa) vs statistical significance (ordinate) The black line indicates no change (fold change = 1), the red lines indicate the 2-fold change threshold, and the blue line in the volcano plots indicates the p value chosen for t-test threshold. A) LGR5 overexpressors vs control cells; B) LGR5 knockdown vs control cells. (TIF) [file pone.0022733.s008.tif]

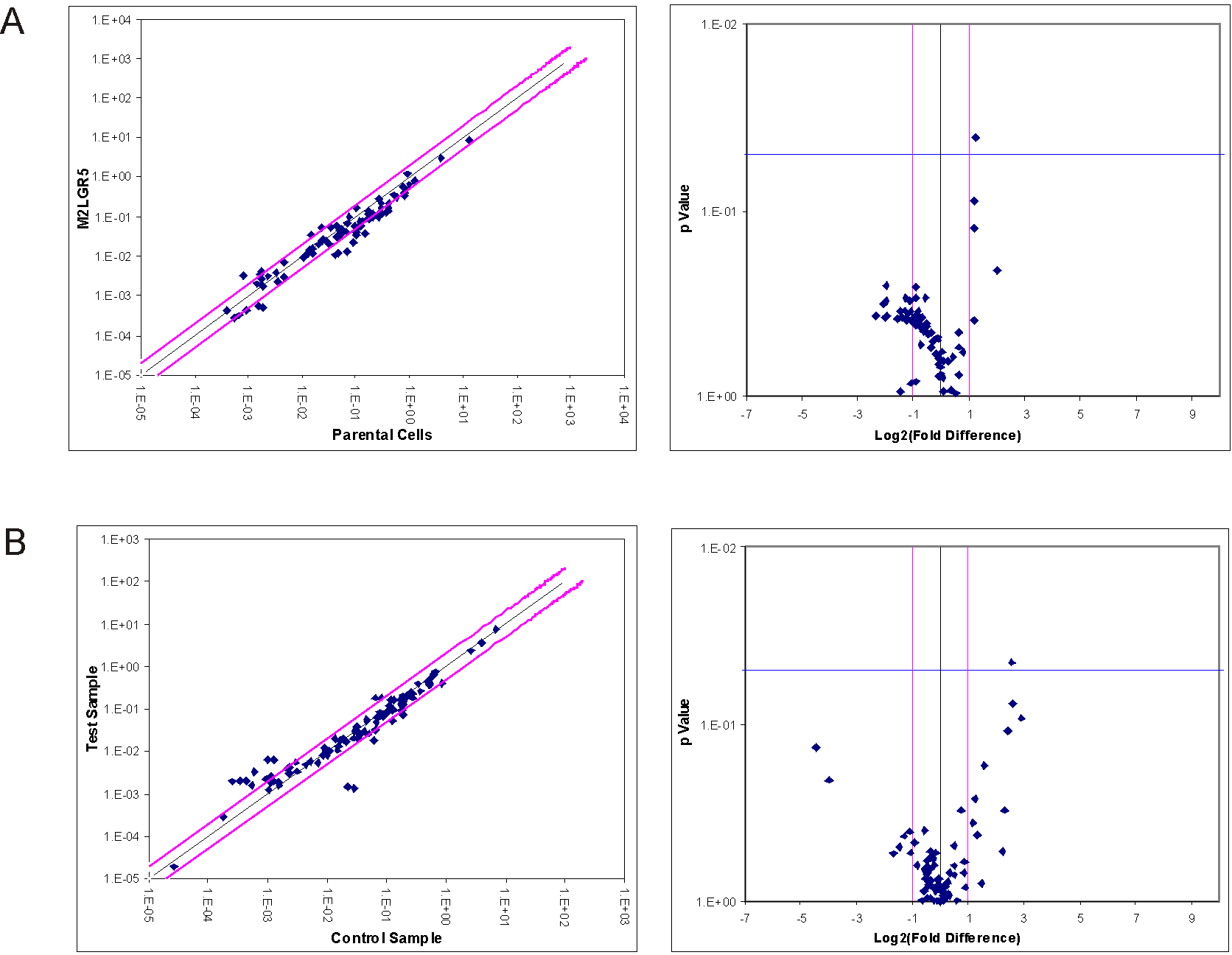

Supplement: Figure S9 — Notch array. mRNA was prepared from LIM1899 untransfected, transfected with empty vectors, with siLGR5 or with pTune/LGR5. Expression of genes in the Notch pathway was determined by qRT-PCR using Superarray plates (SABioscience). The experiments were performed and analyzed as described in Methods. Data are the mean of three independent experiments for each data set. “parental” set includes untransfected cells and cells transfected with empty vectors. Left panels show the correlation in gene expression levels between parental (abscissa) and test (ordinate) samples. Right panels show the “volcano plots” of expression changes (abscissa) vs statistical significance (ordinate) The black line indicates no change (fold change = 1), the red lines indicate the 2-fold change threshold, and the blue line in the volcano plots indicates the p value chosen for t-test threshold. A) LGR5 overexpressors vs control cells; B) LGR5 knockdown vs control cells. (TIF) [file pone.0022733.s009.tif]

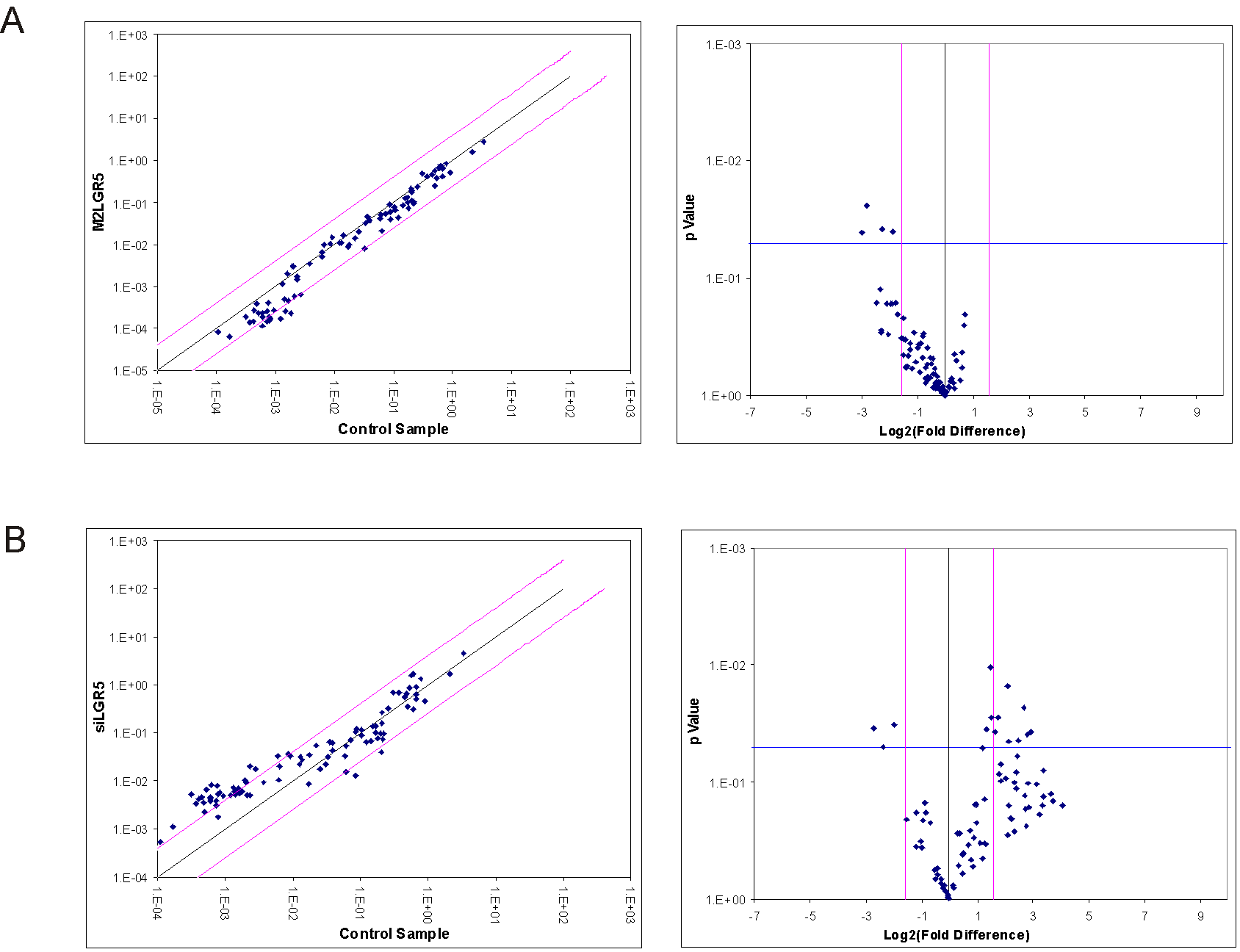

Supplement: Figure S10 — EMT array. mRNA was prepared from LIM1899 untransfected, transfected with empty vectors, with siLGR5 or with pTune/LGR5. Expression of genes in the EMT pathway was determined by qRT-PCR using Superarray plates (SABioscience). The experiments were performed and analyzed as described in Methods. Data are the mean of three independent experiments for each data set. “parental” set includes untransfected cells and cells transfected with empty vectors. Left panels show the correlation in gene expression levels between parental (abscissa) and test (ordinate) samples. Right panels show the “volcano plots” of expression changes (abscissa) vs statistical significance (ordinate) The black line indicates no change (fold change = 1), the red lines indicate the 2-fold change threshold, and the blue line in the volcano plots indicates the p value chosen for t-test threshold. A) LGR5 overexpressors vs control cells; B) LGR5 knockdown vs control cells. (TIF) [file pone.0022733.s010.tif]

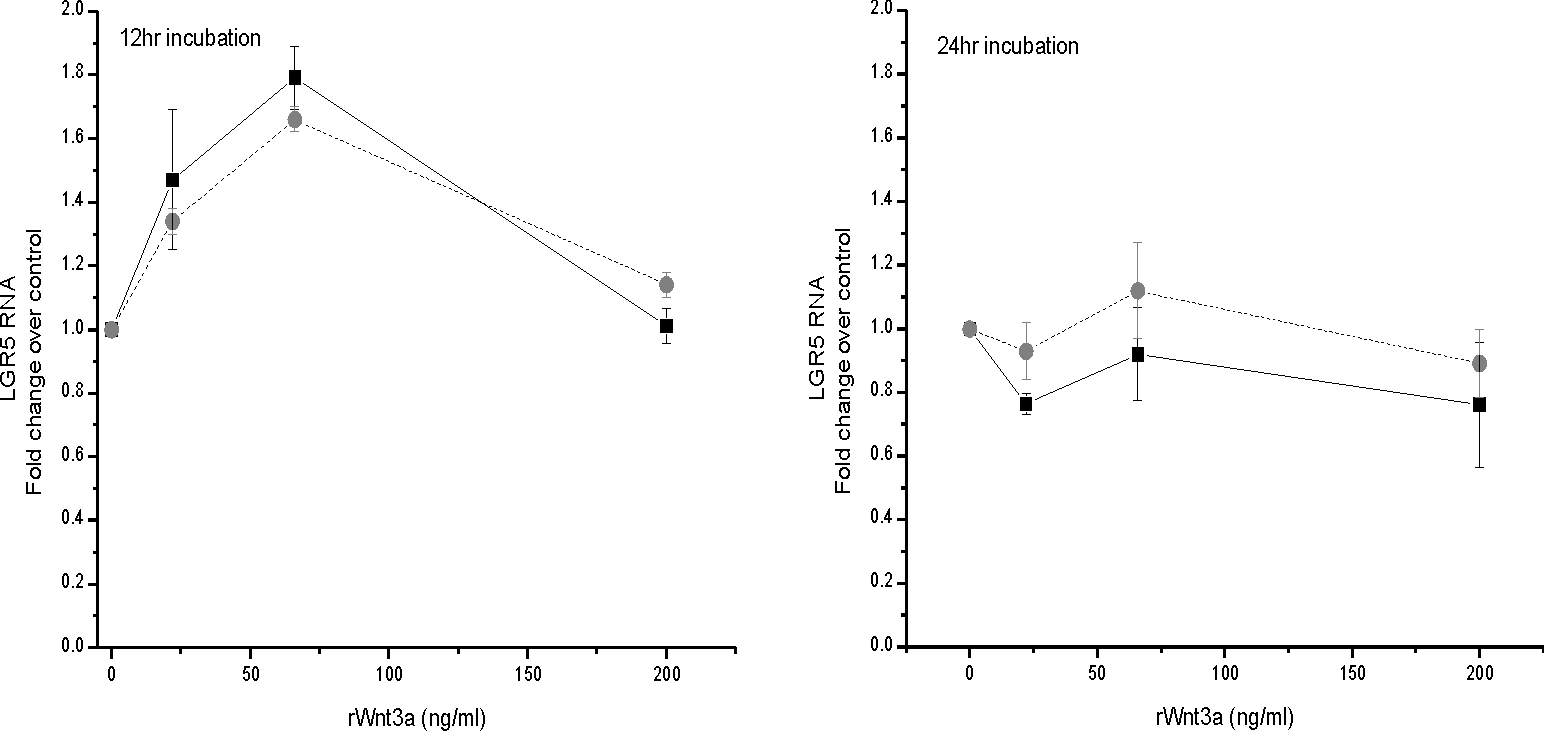

Supplement: Figure S11 — Colorectal cancer cell lines LIM2405 and LIM 2550 plated in 6-well trays were exposed to increasing concentrations of recombinant wnt 3a (0, 22, 66 or 200 ng/ml) and harvested 12 or 24 hrs after addition of the stimulus. mRNA was prepared from each well and the amount of LGR5 message quantitated by qRT-PCR. Duplicate wells were used for each condition, and the experiment was repeated twice. The graphs show the average and standard deviation of duplicate experiments as fold-change in LGR5 expression relative to the internal control (no wnt3a at 12 and 24 hr, respectively). (TIF) [file pone.0022733.s011.tif]
