# Supplementary material for: LGR5 Is a Negative Regulator of Tumourigenicity, Antagonizes Wnt Signalling and Regulates Cell Adhesion in Colorectal Cancer Cell Lines
Source: PLoS One. 2011 Jul 28;6(7):e22733. doi: 10.1371/journal.pone.0022733 (PMC3145754; doi:10.1371/journal.pone.0022733)
Supplement: Table S1 — Wnt Array. Changes in LIM1899 gene expression with upregulation of LGR5. (DOC) [file pone.0022733.s012.doc]

Table S1: Wnt Array. Changes in LIM1899 gene expression with upregulation of LGR5.

| Gene | Fold change over control | p value | Gene | Fold change over control | p value |
| --- | --- | --- | --- | --- | --- |
| AES | -1.41 | 0.389405 | LRP5 | -1.84 | 0.111956 |
| APC | -1.16 | 0.454050 | LRP6 | 1.34 | 0.281107 |
| AXIN1 | -2.43 | 0.101317 | MYC | 1.52 | 0.187403 |
| BCL9 | -1.90 | 0.133270 | NKD1 | 5.17 | N/A |
| BTRC | -1.53 | N/A | NLK | N/A | N/A |
| FZD5 | -2.51 | 0.243529 | PITX2 | 1.17 | 0.733424 |
| CCND1 | -2.20 | 0.253315 | PORCN | 1.63 | 0.346495 |
| CCND2 | -5.58 | 0.010385 | PPP2CA | 1.50 | 0.216236 |
| CCND3 | -1.73 | 0.183385 | PPP2R1A | 1.14 | 0.583417 |
| CSNK1A1 | -1.48 | 0.361491 | PYGO1 | 1.92 | 0.258836 |
| CSNK1D | -1.33 | 0.428881 | RHOU | 1.34 | 0.092441 |
| CSNK1G1 | -1.21 | 0.841476 | SENP2 | 3.40 | 0.084939 |
| CSNK2A1 | 3.68 | 0.188675 | SFRP1 | -1.16 | 0.306634 |
| CTBP1 | 1.45 | 0.151601 | SFRP4 | -1.19 | 0.285075 |
| CTBP2 | 2.16 | 0.088132 | FBXW4 | 1.65 | 0.275101 |
| CTNNB1 | 1.72 | 0.131220 | SLC9A3R1 | 1.32 | 0.140688 |
| CTNNBIP1 | 3.29 | 0.013024 | SOX17 | -1.32 | 0.307780 |
| CXXC4 | 2.19 | 0.267281 | T | 1.95 | 0.266433 |
| DAAM1 | 2.00 | 0.194083 | TCF7 | 2.01 | 0.176294 |
| DIXDC1 | 3.14 | 0.015784 | TCF7L1 | 3.15 | 0.005945 |
| DKK1 | 2.34 | 0.288904 | TLE1 | 2.52 | 0.029975 |
| DVL1 | 3.31 | 0.017453 | TLE2 | 1.89 | 0.214394 |
| DVL2 | -1.46 | 0.153999 | WIF1 | 1.63 | 0.259064 |
| EP300 | 1.46 | 0.288166 | WISP1 | -1.60 | 0.574750 |
| FBXW11 | -1.18 | 0.657456 | WNT1 | 1.04 | 0.294606 |
| FBXW2 | 2.09 | 0.127714 | WNT10A | 1.55 | 0.014935 |
| FGF4 | -6.33 | N/A | WNT11 | 1.81 | 0.074428 |
| FOSL1 | 1.58 | 0.537196 | WNT16 | 1.19 | 0.462855 |
| FOXN1 | -1.00 | 0.289632 | WNT2 | -1.08 | 0.312054 |
| FRAT1 | 2.28 | 0.135092 | WNT2B | 2.47 | 0.044770 |
| FRZB | 1.68 | 0.255715 | WNT3 | 4.30 | 0.000152 |
| FSHB | 1.14 | 0.279865 | WNT3A | 2.09 | 0.252925 |
| FZD1 | 1.33 | 0.277270 | WNT4 | 1.39 | 0.360697 |
| FZD2 | 1.38 | 0.470910 | WNT5A | -2.19 | 0.393730 |
| FZD3 | -1.53 | 0.418678 | WNT5B | -1.25 | 0.337594 |
| FZD4 | 3.29 | 0.167860 | WNT6 | 2.28 | 0.236367 |
| FZD6 | 1.55 | 0.315932 | WNT7A | 1.14 | 0.332034 |
| FZD7 | 1.61 | 0.297697 | WNT7B | -1.37 | N/A |
| FZD8 | 1.57 | 0.015154 | WNT8A | -2.55 | 0.352867 |
| GSK3A | 1.20 | 0.337171 | WNT9A | 1.36 | 0.282120 |
| GSK3B | 1.71 | 0.052045 |  |  |  |
| JUN | 2.53 | 0.039598 |  |  |  |
| KREMEN1 | 1.55 | 0.151739 |  |  |  |
| LEF1 | 1.76 | 0.129535 |  |  |  |
