# Supplementary material for: LGR5 Is a Negative Regulator of Tumourigenicity, Antagonizes Wnt Signalling and Regulates Cell Adhesion in Colorectal Cancer Cell Lines
Source: PLoS One. 2011 Jul 28;6(7):e22733. doi: 10.1371/journal.pone.0022733 (PMC3145754; doi:10.1371/journal.pone.0022733)
Supplement: Table S2 — Wnt array. Changes in LIM1899 gene expression with knockdown of LGR5. (DOC) [file pone.0022733.s013.doc]

| Gene | Fold change over control | p value | Gene | Fold change over control | p value |
| --- | --- | --- | --- | --- | --- |
| AES | 2.20 | 0.446273 | LRP5 | -3.62 | 0.083393 |
| APC | 1.67 | 0.317649 | LRP6 | -1.42 | 0.075664 |
| AXIN1 | 2.26 | 0.072626 | MYC | 1.13 | 0.491826 |
| BCL9 | -1.13 | 0.559628 | NKD1 | -1.09 | N/A |
| BTRC | -1.61 | N/A | NLK | N/A | N/A |
| FZD5 | -2.62 | 0.331613 | PITX2 | -2.46 | 0.125793 |
| CCND1 | -4.31 | 0.256886 | PORCN | -1.93 | 0.246497 |
| CCND2 | -6.91 | 0.026884 | PPP2CA | -1.25 | 0.006909 |
| CCND3 | -31.16 | 0.057216 | PPP2R1A | -1.43 | 0.162867 |
| CSNK1A1 | 4.05 | 0.058332 | PYGO1 | 1.27 | 0.375916 |
| CSNK1D | 2.82 | 0.221242 | RHOU | -1.11 | 0.271851 |
| CSNK1G1 | 4.35 | 0.003853 | SENP2 | -1.50 | 0.217787 |
| CSNK2A1 | -1.73 | 0.122908 | SFRP1 | 1.53 | 0.036017 |
| CTBP1 | -2.05 | 0.038214 | SFRP4 | 2.14 | 0.000484 |
| CTBP2 | -2.52 | 0.047940 | FBXW4 | -1.50 | 0.346133 |
| CTNNB1 | -2.28 | 0.037037 | SLC9A3R1 | -1.08 | 0.700152 |
| CTNNBIP1 | -2.16 | 0.050260 | SOX17 | 2.00 | 0.002325 |
| CXXC4 | 1.01 | 0.689296 | T | 1.66 | 0.014557 |
| DAAM1 | -2.52 | 0.110212 | TCF7 | -2.25 | 0.254948 |
| DIXDC1 | -2.82 | 0.114683 | TCF7L1 | 1.55 | 0.134448 |
| DKK1 | 2.35 | 0.283379 | TLE1 | -1.23 | 0.331419 |
| DVL1 | -1.21 | 0.591654 | TLE2 | -2.38 | 0.016187 |
| DVL2 | -4.76 | 0.029760 | WIF1 | 2.15 | 0.045551 |
| EP300 | -1.80 | 0.080844 | WISP1 | 8.74 | 0.028678 |
| FBXW11 | -1.86 | 0.081628 | WNT1 | 2.23 | 0.003945 |
| FBXW2 | -3.34 | 0.143773 | WNT10A | -2.31 | 0.022239 |
| FGF4 | 3.41 | N/A | WNT11 | -1.99 | 0.109416 |
| FOSL1 | 1.19 | 0.906758 | WNT16 | -2.10 | 0.005827 |
| FOXN1 | 1.69 | 0.106489 | WNT2 | 3.03 | 0.000228 |
| FRAT1 | -3.20 | 0.025601 | WNT2B | -1.07 | 0.629928 |
| FRZB | 2.38 | 0.011037 | WNT3 | 2.99 | 0.038351 |
| FSHB | 2.00 | 0.016016 | WNT3A | 2.17 | 0.000132 |
| FZD1 | -1.74 | 0.029866 | WNT4 | 1.15 | 0.549848 |
| FZD2 | -1.84 | 0.152169 | WNT5A | 3.53 | 0.044501 |
| FZD3 | -5.18 | 0.093035 | WNT5B | 1.56 | 0.005680 |
| FZD4 | 2.55 | 0.018425 | WNT6 | -6.84 | 0.004126 |
| FZD6 | -1.91 | 0.138032 | WNT7A | 1.64 | 0.041883 |
| FZD7 | 2.27 | 0.003818 | WNT7B | 2.20 | N/A |
| FZD8 | -1.64 | 0.078384 | WNT8A | 1.40 | 0.121543 |
| GSK3A | -1.28 | 0.225072 | WNT9A | 1.57 | 0.032437 |
| GSK3B | -1.34 | 0.073897 |  |  |  |
| JUN | -1.51 | 0.086620 |  |  |  |
| KREMEN1 | -2.24 | 0.020864 |  |  |  |
| LEF1 | -4.39 | 0.029796 |  |  |  |

Table S2: Wnt array. Changes in LIM1899 gene expression with knockdown of LGR5.
