# Supplementary material for: LGR5 Is a Negative Regulator of Tumourigenicity, Antagonizes Wnt Signalling and Regulates Cell Adhesion in Colorectal Cancer Cell Lines
Source: PLoS One. 2011 Jul 28;6(7):e22733. doi: 10.1371/journal.pone.0022733 (PMC3145754; doi:10.1371/journal.pone.0022733)
Supplement: Table S3 — Notch Array. Changes in LIM1899 gene expression with overexpression of LGR5. (DOC) [file pone.0022733.s014.doc]

Table S3: Notch Array. Changes in LIM1899 gene expression with overexpression of LGR5.

| Gene | Fold change over control | p value | Gene | Fold change over control | p value |
| --- | --- | --- | --- | --- | --- |
| ADAM10 | -1.93 | 0.367409 | LRP5 | -1.42 | 0.463489 |
| ADAM17 | -1.72 | 0.415648 | MAP2K7 | -2.48 | 0.346718 |
| AES | -2.43 | 0.294343 | MFNG | 1.53 | 0.539371 |
| AXIN1 | -2.20 | 0.305727 | MMP7 | -2.15 | 0.351357 |
| CBL | -1.01 | 0.758827 | MYCL1 | 2.26 | 0.388432 |
| CCND1 | -1.48 | 0.293670 | NCOR2 | -1.85 | 0.257686 |
| CCNE1 | -4.16 | 0.319179 | NEURL | 1.33 | 0.610825 |
| CD44 | -1.57 | 0.438121 | NFKB1 | 2.24 | 0.088914 |
| CDC16 | -1.80 | 0.345598 | NFKB2 | -1.17 | 0.591453 |
| CDKN1A | -1.57 | 0.438055 | NOTCH1 | 1.26 | 0.933993 |
| CFLAR | -2.65 | 0.377005 | NOTCH2 | 1.27 | 0.919945 |
| CHUK | -1.10 | 0.495153 | NOTCH2NL | 1.55 | 0.769377 |
| CTNNB1 | -1.27 | 0.450432 | NOTCH3 | -1.04 | 0.687384 |
| DLL1 | 1.57 | 0.457575 | NOTCH4 | -1.65 | 0.521812 |
| DTX1 | 1.42 | 0.961843 | NR4A2 | -1.99 | 0.389560 |
| EP300 | -2.49 | 0.380330 | NUMB | -2.10 | 0.394043 |
| ERBB2 | -2.73 | 0.348426 | PAX5 | N/A | N/A |
| FIGF | 2.28 | 0.125427 | KAT2B | -1.06 | 0.620451 |
| FOS | -3.03 | 0.382912 | PDPK1 | -1.83 | 0.398693 |
| FOSL1 | -4.02 | 0.252363 | POFUT1 | -3.91 | 0.368500 |
| FZD1 | -1.49 | 0.408832 | PPARG | -1.26 | 0.507872 |
| FZD2 | -1.66 | 0.384271 | PSEN1 | -2.11 | 0.388622 |
| FZD3 | -1.81 | 0.378504 | PSEN2 | -1.08 | 0.775701 |
| FZD4 | -1.08 | 0.669396 | PSENEN | -1.49 | 0.419395 |
| FZD6 | -1.88 | 0.415041 | PTCRA | -2.14 | 0.846768 |
| FZD7 | -5.16 | 0.372042 | RFNG | -1.60 | 0.446871 |
| GBP2 | -1.09 | 0.599899 | RUNX1 | -1.10 | 0.484194 |
| GLI1 | 3.94 | 0.211419 | SEL1L | -1.83 | 0.389834 |
| GSK3B | -1.76 | 0.372473 | SH2D1A | -1.90 | 0.832175 |
| HDAC1 | -1.28 | 0.549423 | SHH | 1.70 | 0.582184 |
| HES1 | -2.81 | 0.350052 | STIL | 1.04 | 0.793645 |
| HEY1 | 2.31 | 0.040259 | SNW1 | -2.31 | 0.357661 |
| HEYL | N/A | N/A | SMO | N/A | N/A |
| HOXB4 | 1.02 | 0.573102 | STAT6 | -1.43 | 0.458310 |
| HR | -4.06 | 0.376222 | SUFU | -1.68 | 0.412318 |
| IFNG | N/A | N/A | TEAD1 | -1.61 | 0.375750 |
| IL17B | N/A | N/A | TLE1 | 1.07 | 0.943959 |
| IL2RA | N/A | N/A | WISP1 | -2.78 | 0.942504 |
| JAG1 | -2.66 | 0.374382 | WNT11 | -1.44 | 0.419770 |
| JAG2 | -1.45 | 0.401169 | ZIC2 | -2.35 | 0.392285 |
| KRT1 | -3.95 | 0.308276 | B2M | N/A | N/A |
| LFNG | -1.88 | 0.295768 | HPRT1 | 1.05 | 0.848237 |
| LMO2 | 1.17 | 0.644206 | RPL13A | 1.28 | 0.219639 |
