# Supplementary material for: LGR5 Is a Negative Regulator of Tumourigenicity, Antagonizes Wnt Signalling and Regulates Cell Adhesion in Colorectal Cancer Cell Lines
Source: PLoS One. 2011 Jul 28;6(7):e22733. doi: 10.1371/journal.pone.0022733 (PMC3145754; doi:10.1371/journal.pone.0022733)
Supplement: Table S4 — Notch Array. Changes in LIM1899 gene expression with knockdown of LGR5. (DOC) [file pone.0022733.s015.doc]

Table S4: Notch Array. Changes in LIM1899 gene expression with knockdown of LGR5.

| Gene | Fold change over control | p value | Gene | Fold change over control | p value |
| --- | --- | --- | --- | --- | --- |
| ADAM10 | -1.05 | 0.664924 | LRP5 | -1.03 | 0.806526 |
| ADAM17 | -1.32 | 0.986574 | MAP2K7 | -1.40 | 0.827481 |
| AES | -1.34 | 0.758226 | MFNG | 1.84 | 0.597245 |
| AXIN1 | 2.27 | 0.656130 | MMP7 | 5.35 | 0.108031 |
| CBL | 1.09 | 0.584681 | MYCL1 | -1.12 | 0.530854 |
| CCND1 | -1.46 | 0.262499 | NCOR2 | -1.17 | 0.621630 |
| CCNE1 | -3.20 | 0.420898 | NEURL | 1.26 | 0.927505 |
| CD44 | -2.11 | 0.670903 | NFKB1 | 1.07 | 0.918306 |
| CDC16 | -1.43 | 0.844378 | NFKB2 | 1.10 | 0.831849 |
| CDKN1A | 1.10 | 0.787181 | NOTCH1 | -1.92 | 0.470275 |
| CFLAR | -1.05 | 0.797357 | NOTCH2 | -21.79 | 0.134706 |
| CHUK | -1.30 | 0.754236 | NOTCH2NL | -15.74 | 0.206902 |
| CTNNB1 | -1.18 | 0.653964 | NOTCH3 | 1.42 | 0.627481 |
| DLL1 | 2.39 | 0.835268 | NOTCH4 | 1.06 | 0.843003 |
| DTX1 | 2.49 | 0.528410 | NR4A2 | -1.20 | 0.568089 |
| EP300 | -1.46 | 0.991115 | NUMB | -1.36 | 0.689353 |
| ERBB2 | -1.17 | 0.427512 | PAX5 | 1.30 | N/A |
| FIGF | 1.17 | 0.691654 | KAT2B | -1.04 | 0.988588 |
| FOS | 2.78 | 0.947831 | PDPK1 | 1.03 | 0.882865 |
| FOSL1 | -1.26 | 0.171005 | POFUT1 | -1.49 | 0.870009 |
| FZD1 | -1.44 | 0.590400 | PPARG | 1.43 | 0.486102 |
| FZD2 | 1.89 | 0.518446 | PSEN1 | 1.21 | 0.906172 |
| FZD3 | -2.07 | 0.868544 | PSEN2 | -1.07 | 0.986007 |
| FZD4 | 1.52 | 0.305189 | PSENEN | -1.16 | 0.986706 |
| FZD6 | -2.45 | N/A | PTCRA | 4.73 | 0.525089 |
| FZD7 | 1.27 | 0.567268 | RFNG | 1.21 | 0.773146 |
| GBP2 | 1.12 | 0.807310 | RUNX1 | -1.43 | 0.741769 |
| GLI1 | 3.01 | N/A | SEL1L | 1.06 | 0.929131 |
| GSK3B | -1.41 | N/A | SH2D1A | 5.94 | 0.044544 |
| HDAC1 | -1.29 | N/A | SHH | -1.07 | 0.992450 |
| HES1 | -1.09 | 0.738860 | STIL | -1.41 | 0.703627 |
| HEY1 | 1.65 | 0.620438 | SNW1 | -1.36 | 0.951281 |
| HEYL | 1.05 | 0.307032 | SMO | -1.39 | N/A |
| HOXB4 | -1.29 | 0.492773 | STAT6 | 1.15 | 0.826624 |
| HR | -1.33 | 0.688370 | SUFU | -1.28 | 0.954539 |
| IFNG | 1.14 | 0.091880 | TEAD1 | 1.06 | 0.857308 |
| IL17B | 1.56 | 0.664924 | TLE1 | 1.40 | 0.710858 |
| IL2RA | 1.15 | 0.986574 | WISP1 | 6.01 | 0.077007 |
| JAG1 | -1.04 | 0.758226 | WNT11 | -1.02 | 0.994561 |
| JAG2 | -1.78 | 0.656130 | ZIC2 | -1.52 | 0.984236 |
| KRT1 | 4.93 | 0.584681 | B2M | -1.09 | N/A |
| LFNG | -2.78 | 0.262499 | HPRT1 | -1.90 | 0.170938 |
| LMO2 | 1.81 | 0.420898 | RPL13A | 1.01 | 0.974237 |
