# Supplementary material for: LGR5 Is a Negative Regulator of Tumourigenicity, Antagonizes Wnt Signalling and Regulates Cell Adhesion in Colorectal Cancer Cell Lines
Source: PLoS One. 2011 Jul 28;6(7):e22733. doi: 10.1371/journal.pone.0022733 (PMC3145754; doi:10.1371/journal.pone.0022733)
Supplement: Table S5 — EMT Array. Changes in LIM1899 gene expression with overexpression of LGR5. (DOC) [file pone.0022733.s016.doc]

Table S5: EMT Array. Changes in LIM1899 gene expression with overexpression of LGR5.

| Gene | Fold change over control | | p value | Gene | Fold change over control | p value |
| --- | --- | --- | --- | --- | --- | --- |
| AHNAK | -1.47 | | 7.0E-01 | NOTCH1 | -2.19 | 3.0E-01 |
| AKT1 | -1.85 | | 3.6E-01 | NUDT13 | -1.95 | 3.7E-01 |
| BMP1 | -3.89 | | 1.7E-01 | OCLN | 1.16 | 7.2E-01 |
| BMP7 | -2.40 | | 3.6E-01 | PDGFRB | -2.84 | 3.3E-01 |
| CALD1 | -2.81 | | 2.2E-01 | PLEK2 | 1.04 | 9.4E-01 |
| CAMK2N1 | -1.13 | | 9.0E-01 | PPPDE2 | 1.02 | 9.5E-01 |
| CAV2 | 1.00 | | 9.9E-01 | PTK2 | -1.06 | 8.5E-01 |
| CDH1 | -1.56 | | 5.4E-01 | PTP4A1 | 1.10 | 8.4E-01 |
| CDH2 | -2.64 | | 5.9E-01 | RAC1 | 1.18 | 7.3E-01 |
| COL1A2 | -5.03 | | 2.9E-01 | RGS2 | -1.75 | 4.7E-01 |
| COL3A1 | -1.64 | | 7.8E-01 | SERPINE1 | 1.11 | 8.5E-01 |
| COL5A2 | 1.50 | | 5.9E-01 | SIP1 | -1.03 | 9.1E-01 |
| CTNNB1 | -1.76 | | 3.1E-01 | SMAD2 | 1.32 | 5.0E-01 |
| DSC2 | -1.20 | | 7.7E-01 | SNAI1 | 1.53 | 4.3E-01 |
| DSP | 1.25 | | 4.4E-01 | SNAI2 | -1.13 | 8.9E-01 |
| EGFR | -1.59 | | 4.0E-01 | SNAI3 | -1.38 | 6.6E-01 |
| ERBB3 | -1.44 | | 5.4E-01 | SOX10 | -3.74 | 1.7E-01 |
| ESR1 | -1.91 | | 6.4E-01 | SPARC | -7.02 | 2.4E-02 |
| F11R | -1.98 | | 3.9E-01 | SPP1 | -2.28 | 6.0E-01 |
| FGFBP1 | -2.10 | | 5.2E-01 | STAT3 | -1.75 | 3.0E-01 |
| FN1 | -1.26 | | 8.7E-01 | STEAP1 | -1.21 | 8.2E-01 |
| FOXC2 | -2.85 | | 4.5E-01 | TCF3 | 1.00 | 1.0E+00 |
| FZD7 | -1.56 | | 7.5E-01 | TCF4 | -1.29 | 5.9E-01 |
| GNG11 | -2.65 | | 5.8E-01 | TFPI2 | -2.60 | 5.6E-01 |
| GSC | -1.37 | | 8.5E-01 | TGFB1 | -2.70 | 3.4E-01 |
| GSK3B | -1.35 | | 4.9E-01 | TGFB2 | 1.63 | 2.0E-01 |
| IGFBP4 | -1.66 | | 5.8E-01 | TGFB3 | -1.07 | 9.4E-01 |
| IL1RN | 1.25 | | 8.7E-01 | TIMP1 | 1.60 | 2.5E-01 |
| ILK | -1.45 | | 4.8E-01 | TMEFF1 | 1.14 | 7.6E-01 |
| ITGA5 | 1.23 | | 7.8E-01 | TMEM132A | -2.97 | 3.2E-01 |
| ITGAV | -1.37 | | 4.8E-01 | TSPAN13 | -1.23 | 6.9E-01 |
| ITGB1 | -1.09 | | 8.6E-01 | TWIST1 | -4.97 | 2.8E-01 |
| JAG1 | -1.14 | | 7.7E-01 | VCAN | -4.14 | 3.0E-01 |
| KRT14 | -4.33 | | 1.7E-01 | VIM | -1.26 | 8.4E-01 |
| KRT19 | -1.31 | | 7.9E-01 | VPS13A | -1.03 | 9.5E-01 |
| KRT7 | -3.63 | | 4.0E-02 | WNT11 | -1.30 | 6.6E-01 |
| MAP1B | 1.45 | | 7.5E-01 | WNT5A | -4.88 | 3.8E-02 |
| MITF | -1.65 | | 7.2E-01 | WNT5B | -3.26 | 2.1E-01 |
| MMP2 | -5.67 | | 1.6E-01 | ZEB1 | -3.45 | 1.6E-01 |
| MMP3 | -5.09 | | 1.2E-01 | ZEB2 | -2.54 | 4.6E-01 |
| MMP9 | -7.91 | | 4.1E-02 |  |  |  |
| MSN | -1.59 | | 7.0E-01 |  |  |  |
| MST1R | -2.41 | | 4.0E-01 |  |  |  |
| NODAL | -1.23 | 7.7E-01 | |  |  |  |
