# Supplementary material for: LGR5 Is a Negative Regulator of Tumourigenicity, Antagonizes Wnt Signalling and Regulates Cell Adhesion in Colorectal Cancer Cell Lines
Source: PLoS One. 2011 Jul 28;6(7):e22733. doi: 10.1371/journal.pone.0022733 (PMC3145754; doi:10.1371/journal.pone.0022733)
Supplement: Table S6 — EMT Array. Changes in LIM1899 gene expression with knockdown of LGR5. (DOC) [file pone.0022733.s017.doc]

Table S6: EMT Array. Changes in LIM1899 gene expression with knockdown of LGR5.

| Gene | Fold change over control | | p value | Gene | Fold change over control | p value |
| --- | --- | --- | --- | --- | --- | --- |
| AHNAK | 1.37 | | 6.1E-01 | NOTCH1 | -6.66 | 3.4E-02 |
| AKT1 | -5.21 | | 5.0E-02 | NUDT13 | -1.99 | 3.6E-01 |
| BMP1 | -1.39 | | 6.7E-01 | OCLN | 2.82 | 1.1E-02 |
| BMP7 | -2.87 | | 2.1E-01 | PDGFRB | 5.30 | 1.1E-01 |
| CALD1 | 4.32 | | 1.6E-01 | PLEK2 | 1.27 | 5.2E-01 |
| CAMK2N1 | 1.78 | | 5.3E-01 | PPPDE2 | 1.23 | 2.7E-01 |
| CAV2 | 1.65 | | 2.6E-01 | PTK2 | 1.28 | 2.7E-01 |
| CDH1 | -1.01 | | 9.8E-01 | PTP4A1 | 1.84 | 3.0E-01 |
| CDH2 | 9.50 | | 1.9E-01 | RAC1 | 1.40 | 4.0E-01 |
| COL1A2 | 6.72 | | 1.7E-01 | RGS2 | 1.95 | 2.2E-01 |
| COL3A1 | 16.59 | | 1.6E-01 | SERPINE1 | 5.56 | 4.4E-02 |
| COL5A2 | 5.38 | | 6.0E-02 | SIP1 | 1.09 | 7.6E-01 |
| CTNNB1 | -1.95 | | 2.1E-01 | SMAD2 | 1.86 | 1.5E-01 |
| DSC2 | -1.08 | | 8.6E-01 | SNAI1 | 3.16 | 3.7E-02 |
| DSP | 2.86 | | 2.8E-02 | SNAI2 | 5.31 | 8.2E-02 |
| EGFR | 1.11 | | 7.9E-01 | SNAI3 | 2.13 | 3.3E-01 |
| ERBB3 | 1.59 | | 3.4E-01 | SOX10 | 2.43 | 3.3E-01 |
| ESR1 | 10.18 | | 1.6E-01 | SPARC | 4.11 | 9.2E-02 |
| F11R | -1.35 | | 5.5E-01 | SPP1 | 12.59 | 1.3E-01 |
| FGFBP1 | -2.27 | | 3.6E-01 | STAT3 | -1.63 | 2.2E-01 |
| FN1 | 7.38 | | 1.6E-01 | STEAP1 | 2.43 | 1.4E-01 |
| FOXC2 | 10.38 | | 7.9E-02 | TCF3 | -1.03 | 9.2E-01 |
| FZD7 | 2.53 | | 3.5E-02 | TCF4 | -1.23 | 7.3E-01 |
| GNG11 | 6.77 | | 2.4E-01 | TFPI2 | 13.10 | 1.4E-01 |
| GSC | 5.03 | | 2.7E-01 | TGFB1 | -1.85 | 1.5E-01 |
| GSK3B | -1.12 | | 7.5E-01 | TGFB2 | 3.66 | 7.0E-02 |
| IGFBP4 | -2.04 | | 3.2E-01 | TGFB3 | 2.51 | 3.4E-01 |
| IL1RN | 4.40 | | 4.4E-02 | TIMP1 | 2.33 | 5.2E-02 |
| ILK | -1.83 | | 1.8E-01 | TMEFF1 | 1.96 | 1.5E-01 |
| ITGA5 | 4.26 | | 1.5E-02 | TMEM132A | -3.98 | 3.2E-02 |
| ITGAV | -1.02 | | 9.6E-01 | TSPAN13 | -1.17 | 8.1E-01 |
| ITGB1 | 1.37 | | 4.1E-01 | TWIST1 | 4.25 | 2.8E-01 |
| JAG1 | -1.33 | | 6.1E-01 | VCAN | 2.31 | 4.5E-01 |
| KRT14 | 6.45 | | 1.3E-01 | VIM | 4.72 | 2.0E-01 |
| KRT19 | -1.21 | | 6.7E-01 | VPS13A | 1.26 | 5.2E-01 |
| KRT7 | 3.46 | | 8.5E-02 | WNT11 | -1.44 | 5.6E-01 |
| MAP1B | 4.63 | | 2.0E-01 | WNT5A | 7.02 | 3.9E-02 |
| MITF | 10.43 | | 1.3E-01 | WNT5B | 7.73 | 3.7E-02 |
| MMP2 | 3.66 | | 9.8E-02 | ZEB1 | 5.00 | 1.0E-01 |
| MMP3 | 6.43 | | 2.3E-02 | ZEB2 | 7.17 | 1.0E-01 |
| MMP9 | 3.40 | | 2.8E-02 |  |  |  |
| MSN | 8.75 | | 1.0E-01 |  |  |  |
| MST1R | -2.27 | | 1.8E-01 |  |  |  |
| NODAL | 1.71 | 4.6E-01 | |  |  |  |
