# Supplementary material for: LGR5 Is a Negative Regulator of Tumourigenicity, Antagonizes Wnt Signalling and Regulates Cell Adhesion in Colorectal Cancer Cell Lines
Source: PLoS One. 2011 Jul 28;6(7):e22733. doi: 10.1371/journal.pone.0022733 (PMC3145754; doi:10.1371/journal.pone.0022733)
Supplement: Table S7 — Primer list for quantitative real-time PCR. (DOC) [file pone.0022733.s018.doc]

Table S7: Primer list for quantitative real-time PCR

| Gene name | Forward primer | Reverse primer |
| --- | --- | --- |
| GAPDH | CAATGACCCCTTCATTGACC | TGATGACAAGCTTCCCGTTC |
| Lgr5 | CTTCCAACCTCAGCGTCTTC | TTTCCCGCAAGACGTAACTC |
| MSI 1 | GGGGGTGGATAAAGTGCTG | CGTTCGAGTCACCATCTTGG |
| Lgr6 | ACCTGGACCTCAGCATGAAC | GAATGCTTGTCCTGGGATGT |
| B2M | TTCTGGCCTGGAGGCTATC | TCAATGTCGGATGGATGAAA |
